# CKX Inhibitors: From Shoot Tip Micropropagation to Rooted Plantlet

**DOI:** 10.3390/plants15213293

**Published:** 2026-10-25

**Authors:** Florin Adrian Huiban, Vladislava Galović, Tatjana Vujović, Jaroslav Nisler, Saša Orlović, Branislav Kovačević, Jovana Šućur Elez, Marina Crnković, Camelia Tulcan, Dorin Camen

**Affiliations:** 1 Doctoral School Engineering of Plant and Animal Resources, University of Life Sciences “King Mihai I” from Timisoara, 300645 Timisoara, Romania; florin.huiban@usvt.ro (F.A.H.); dorincamen@usvt.ro (D.C.); 2 Research Institute for Biosecurity and Bioengineering, University of Life Sciences “King Mihai I” from Timisoara, 300645 Timisoara, Romania; 3 Faculty of Horticulture, Forestry and Plant Biotechnologies, University of Life Sciences “King Mihai I” from Timisoara, 300645 Timisoara, Romania; 4 Institute of Lowland Forestry and Environment, University of Novi Sad, 21000 Novi Sad, Serbia; sasao@uns.ac.rs (S.O.); branek@uns.ac.rs (B.K.); 5 Institute for Fruit Growing, Kralja Petra I, 32102 Čačak, Serbia; tvujovic@institut-cacak.org; 6 Institute of Experimental Botany of Czech Academy of Sciences, Videnska 1083, 142 20 Prague, Czech Republic; jaroslav.nisler@gmail.com; 7 Faculty of Agriculture, University of Novi Sad, Trg Dositeja Obradovića 8, 21000 Novi Sad, Serbia; jovana.sucur@polj.edu.rs (J.Š.E.); marina.crnkovic@polj.uns.ac.rs (M.C.)

**Keywords:** cytokinin oxidase/dehydrogenase inhibitors, *Populus alba*, micropropagation, adventitious rooting

## Abstract

Cytokinin homeostasis has an indispensable role in plant developmental processes and is regulated, in part, by cytokinin oxidase/dehydrogenases (CKXs), which irreversibly deactivate cytokinins. Recently developed CKX inhibitors offer a promising approach for modulating endogenous cytokinin activity and overcoming limitations in plant regeneration and micropropagation. In this study, we evaluated the effects of two diphenylurea-based CKX inhibitors, compounds **52** and **77**, on the in vitro clonal propagation of two *Populus alba* clones, Villafranca and L-12. The compounds were applied at concentrations of 1 µM and 0.1 µM, either alone or in combination with 1 µM 2-isopentenyladenine (2iP) or 1 µM 6-benzylaminopurine (BAP). After 48 days, we analyzed multiplication parameters, biomass, pigment content, and biochemical parameters. For Villafranca, compound **52** at 0.1 µM yielded the highest multiplication rate (5.57), and media containing only inhibitors had a rooting percentage exceeding 80%. At 0.1 µM, the inhibitors tended to increase total chlorophyll content, but only in Villafranca. For L-12, multiplication rate was lower, showing no major differences across most formulations. L-12 exhibited a higher content of phenolic compounds and flavonoids, as well as greater antioxidant capacity, but exhibited a lower rooting rate compared to Villafranca. Multiplication and rooting rate on the same medium, in the absence of exogenous cytokinins, were achieved differently in L-12 and Villafranca clones, indicating a genotype-dependent response.

## 1. Introduction

Because of severe climate change, to save overall plant production in forest and crop sectors, it was necessary to direct extensive research on ways of improving and controlling plant growth regulators (PGRs). Among the common PGRs, cytokinins play an indispensable function in overall morphogenesis [1,2,3,4]. It has been revealed that appropriate levels of cytokinin are necessary because they govern the complex signaling network [5] that directs morphogenic responses, which are essential physiological and regulatory responses in different cell types [6]. They are involved in stem elongation and cell differentiation, but they also play an important role in plant development, nutrient uptake, and stress responses [7]. Therefore, cytokinins can be employed as potential agrochemicals [8,9]. Besides these, another way to increase cytokinin levels is to inhibit cytokinin degradation [10]. Chemically, cytokinins are derived from adenine and are recognized by the isoprenoid or aromatic chain attached to the N_6_ position of the purine ring [11]. They have a main regulatory function in in vitro cultures, as they are responsible for both shoot multiplication and differentiation [12]. The level of active cytokinins in a tissue is largely controlled by the enzyme cytokinin oxidase/dehydrogenase (CKX). These cleave the side chain at the N_6_ position and convert the hormone to adenine or adenosine, and the reaction is irreversible [13]. The higher the CKX activity, the less cytokinin remains available for growth. For this reason, blocking these enzymes with chemical inhibitors has become an interesting direction of work for forestry, horticulture, agriculture, and the pharmaceutical industry, especially through micropropagation, which allows the rapid multiplication of valuable genotypes [14]. Moreover, the application of these inhibitors can be considered among the best alternatives to genetic modification for overcoming recalcitrance and saving time in micropropagation in in vitro cultures of such species [10].

White poplar (*Populus alba* L.) is a forest species with a wide range of uses, from biomass and bio-energy production to afforestation and soil conservation [14]. The clones Villafranca and L-12, used in the present work, were developed from positive selection for straight trunk, vigorous growth, and tolerance to biotic and abiotic stress, and their behavior has already been followed in studies on drought response and antioxidant capacity [15]. Clonal multiplication can be efficiently conducted via micropropagation, but this method encounters a well-recognized problem: recalcitrance. Some woody genotypes respond poorly to standard culture protocols, with low multiplication rates or explants that do not develop normally [16].

In poplar, as in most woody species, rooting remains the most difficult step [17]. The in vitro recalcitrance of woody species manifests as a series of successive bottlenecks. During the multiplication phase, many genotypes exhibit a low rate of axillary proliferation, and the response to the same hormonal regimen varies significantly among clones [16,18]. Exogenous cytokinins inhibit adventitious root formation [19], necessitating a separate rooting stage on an auxin-containing medium, a stage that remains sensitive in the case of poplar [17].

A relatively new direction is the use of CKX inhibitors, substances that block the enzyme and thus maintain a higher level of active cytokinin in the tissue. A good part of them, including the compounds evaluated here, belong to the diphenylurea class [9]. Results obtained in recent years recommend them as tools with real potential in plant biotechnology, both for regeneration and multiplication [20,21].

**Figure 1:**
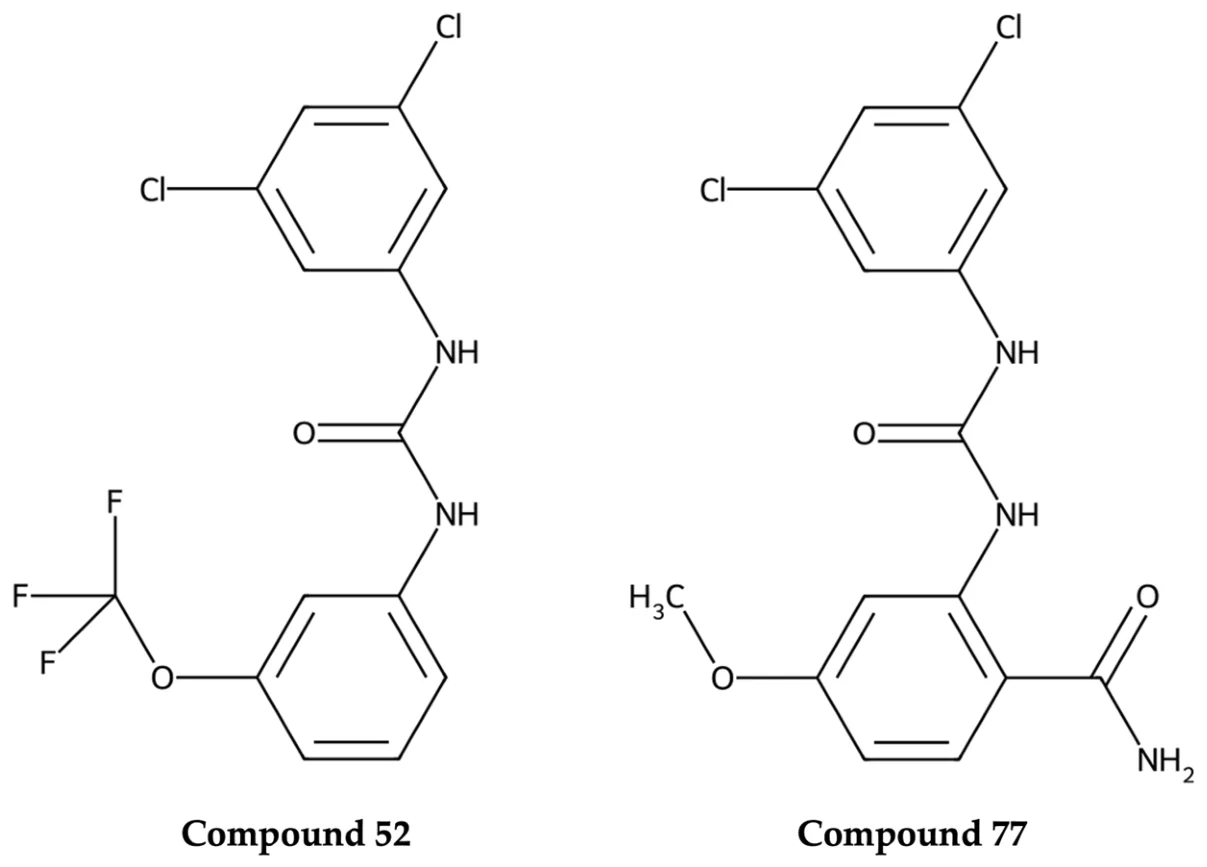
Structural formula of the CKX inhibitors used in the study: compound **52** (1-(3,5-Dichlorophenyl)-3-(3-(trifluoromethoxy)phenyl)urea) and compound **77** (2-(3-(3,5-Dichlorophenyl)ureido)-4-methoxybenzamide).

Two of the compounds with the best profiles described so far are compound **52** (1-(3,5-Dichlorophenyl)-3-(3-(trifluoromethoxy)phenyl)urea) a halogenated diphenylurea containing one 3,5-dihlorofenil, one 3-(trifluorometoksi)fenil, and one central urea group and compound **77** (2-(3-(3,5-Dichlorophenyl)ureido)-4-methoxybenzamide) has additionally modified structure with benzamide group, which led to highly advanced inhibition (Figure 1) [18,20,21]. Both have been previously tested on several herbaceous and ornamental species, including tobacco, *Lobelia*, *Plectranthus*, *Lavandula*, *Sempervivum* and *Drosera*, and the results varied widely from one species to another [20,21]. Compound **52**, applied together with 2iP, clearly improved shoot formation in tobacco and *Lobelia*. Compound **77** gave good results in *Lobelia* and *Plectranthus*, but almost null in *Lavandula* and *Sempervivum* [20]. These differences indicate that the effectiveness of CKX inhibitors depends on the treated species, so each species of interest must be tested separately. Another aspect that matters for practical application is safety: tests on the nematode *Caenorhabditis elegans* showed that the two compounds behave differently, with compound **77** not being toxic to the tested organism, while compound **52** showed a certain degree of toxicity [21].

Until now, research on CKX inhibitors has focused almost exclusively on model plants or herbaceous species, and woody species of forestry interest have remained outside these tests [20,21]. However, the recalcitrance to in vitro culture is more pronounced in these species, so it is precisely there that such a tool would be more useful.

The aim of this study was to evaluate the effect of compounds **52** and **77** on in vitro micropropagation in two clones of *Populus alba*, Villafranca and L-12, through morphological, physiological, and biochemical parameters measured on the treated explant. The compounds were tested at 0.1 and 1 µM with and without exogenous cytokinin 2iP or BAP and compared against two control media containing only cytokinin. After 48 days, the explant multiplication, growth, rooting, photosynthetic pigment content, phenolic compounds, and antioxidant capacity were evaluated. The hypothesis we started from was that these CKX inhibitors can increase the efficiency of poplar micropropagation and improve both the multiplication rate and the quality of the shoots obtained, and the results could pave the way for other forest species of economic interest.

## 2. Results

Analyzing novel CKX inhibitors in plants, compounds **77** and **52** were tested for their ability to positively affect plant growth, development, and the induction of shoot formation in the presence or absence of exogenous cytokinins 2iP and BAP in two different white poplar clones. The efficiency of compound **77** was compared with that of compound **52** for several morphological, photosynthetic pigment content, phenolic compound content, and antioxidant capacity parameters.

### 2.1. Morphometric and Photosynthetic Parameters

The effect of the treatments on morphometric parameters and photosynthetic pigment content was evaluated using two-way factorial analysis of variance and Duncan’s test at a significance level of α = 0.05. The 14 formulations compared include the two CKX inhibitors, each at two concentrations (0.1 and 1 µM) applied either alone or in combination with 1 µM 2iP or 1 µM BAP, along with two control media containing 1 µM 2iP and 1 µM BAP, respectively. The parameters monitored were the height of the dominant shoot, the multiplication rate and percentage, moisture content, shoot fresh and dry mass, as well as the content of chlorophyll a, chlorophyll b, carotenoids, and total chlorophyll (a+b).

**Figure 2:**
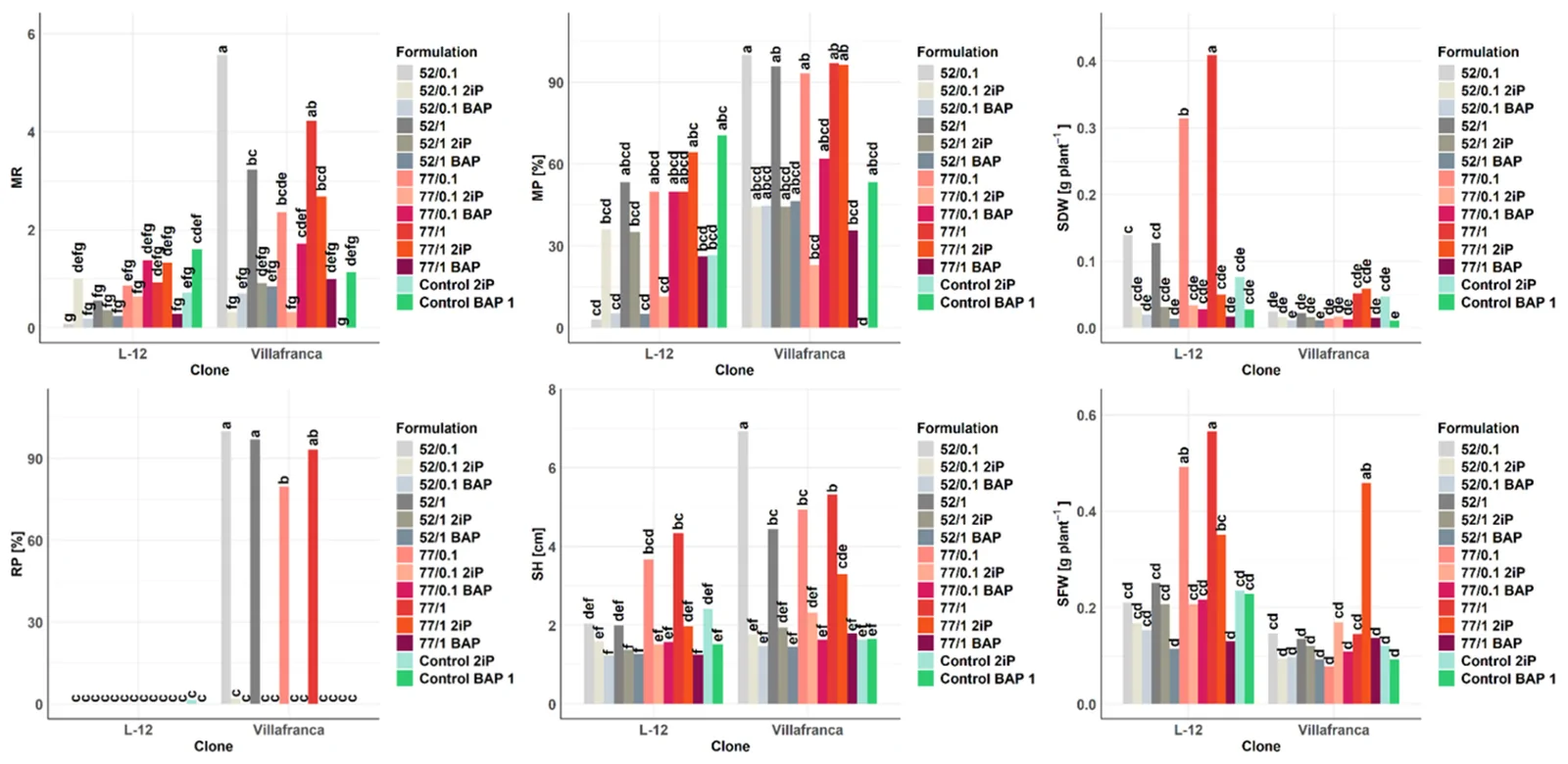
Results of Duncan’s test for morphometric parameters in white poplar clones (L-12 and Villafranca) after 48 days in vitro. MR—multiplication rate; MP—multiplication percentage (%); SH—shoot height (cm); SFW—shoot fresh weight (g plant^−1^); SDW—shoot dry weight (g plant^−1^); RP—rooting percentage (%). Different lowercase letters above bars indicate statistically significant differences among clone × formulation combinations (Duncan’s multiple range test, *p* ≤ 0.05). Treatment labels: The first part of the label represents the CKX inhibitor, the second its medium concentration in µM, and the third part, 2iP and BAP in medium at a concentration of 1 µM. Control media contained no CKX inhibitor.

The greatest differences were found for shoot height, where L-12 on 52/0.1 clearly stands out with 6.9 cm, while all the other combinations remain below 5.5 cm (Figure 2). The multiplication rate follows an opposite pattern: Villafranca on 52/0.1 reaches the highest value (~5.6), while the same formulation on L-12 is the lowest on the entire chart (~0.1). The multiplication percentage is highest in several Villafranca formulations (52/0.1, 52/1, 77/0.1, 77/1, 77/1 2iP), all close, between 93 and 100%. For shoot dry mass, only two combinations stand out: 77/1 (~0.41 g) and 77/0.1 (~0.315 g) in Villafranca. The rest of the columns are small and similar. Rooting in L-12 was sporadic, while at Villafranca four formulations (52/0.1, 52/1, 77/1, 77/0.1) exceeded 80%. The fresh mass is also highest at Villafranca on 77/1 (~0.57 g).

Considering the average of both clones (main effect of the formulation) regarding the treatments of the formulation factor, the highest SH was found at concentrations of 0.1 and 1 µM of compound **77** (77/0.1 and 77/1, respectively) and 0.1 µM compound **52** (52/0.1), significantly higher than on control media with BAP or 2iP alone. The highest multiplication rate was found on 77/1 and the highest multiplication percentage on 77/1 2iP media. That was significantly higher than MR and MP achieved on Control 2iP, but not higher than MR and MP on Control BAP. The highest shoot fresh weight (SFW) was recorded on 77/1 2iP and 77/1 (0.405 and 0.356 g plant-1, respectively), while the highest shoot dry mass was achieved on 77/1 and 77/0.1 (0.231 and 0.163 g plant-1, respectively). The highest content of Car and Chl a+b was recorded on 77/1 2iP, while the highest Chl b content was on 52/0.1 and 77/1. The main effect of the formulation on Chl a content was not significant (Table 1); the numerical differences between formulations for this parameter are not interpreted as treatment effects. Since the clone × formulation interaction was significant for the majority of parameters (Table 1), these mean effects must be interpreted in conjunction with the response of each clone, presented below.

**Figure 3:**
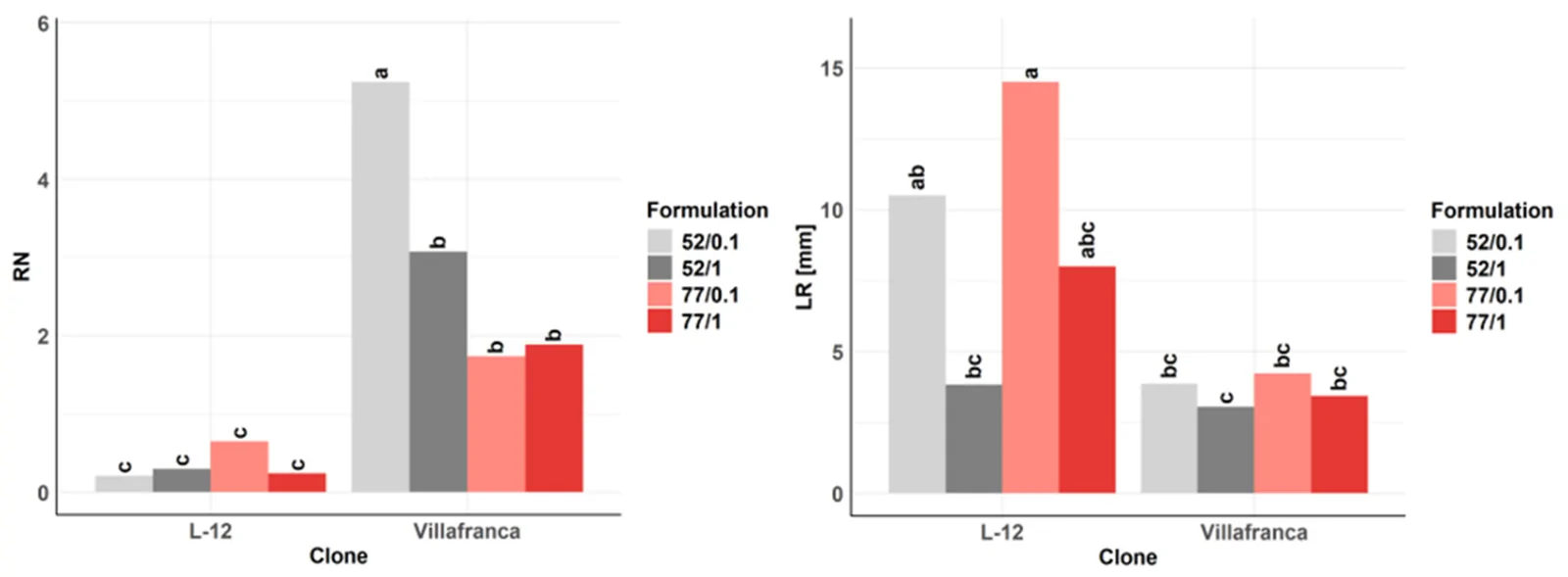
Results of Duncan’s test for number of roots (RN) and length of the longest root (LR [mm]) in white poplar clones (L-12 and Villafranca) after 48 days in vitro. Different lowercase letters above bars indicate statistically significant differences among Clone × Formulation combinations (Duncan’s multiple range test, *p* ≤ 0.05). Treatment labels: The first part of the label represents the CKX inhibitor and the second its medium concentration in µM.

Rooting occurred almost exclusively on media lacking exogenous cytokinin and supplemented only with the CKX inhibitor (Figure 2). On these media, clone Villafranca exhibited a high rooting rate, with the highest number of roots observed on the medium containing compound **52** at 0.1 µM. For clone L-12, rooting was sporadic and produced few roots, though these were longer than those of clone Villafranca (Figure 3; Table S3). On media containing 2iP or BAP, including the controls, rooting was virtually absent in both clones.

**Figure 4:**
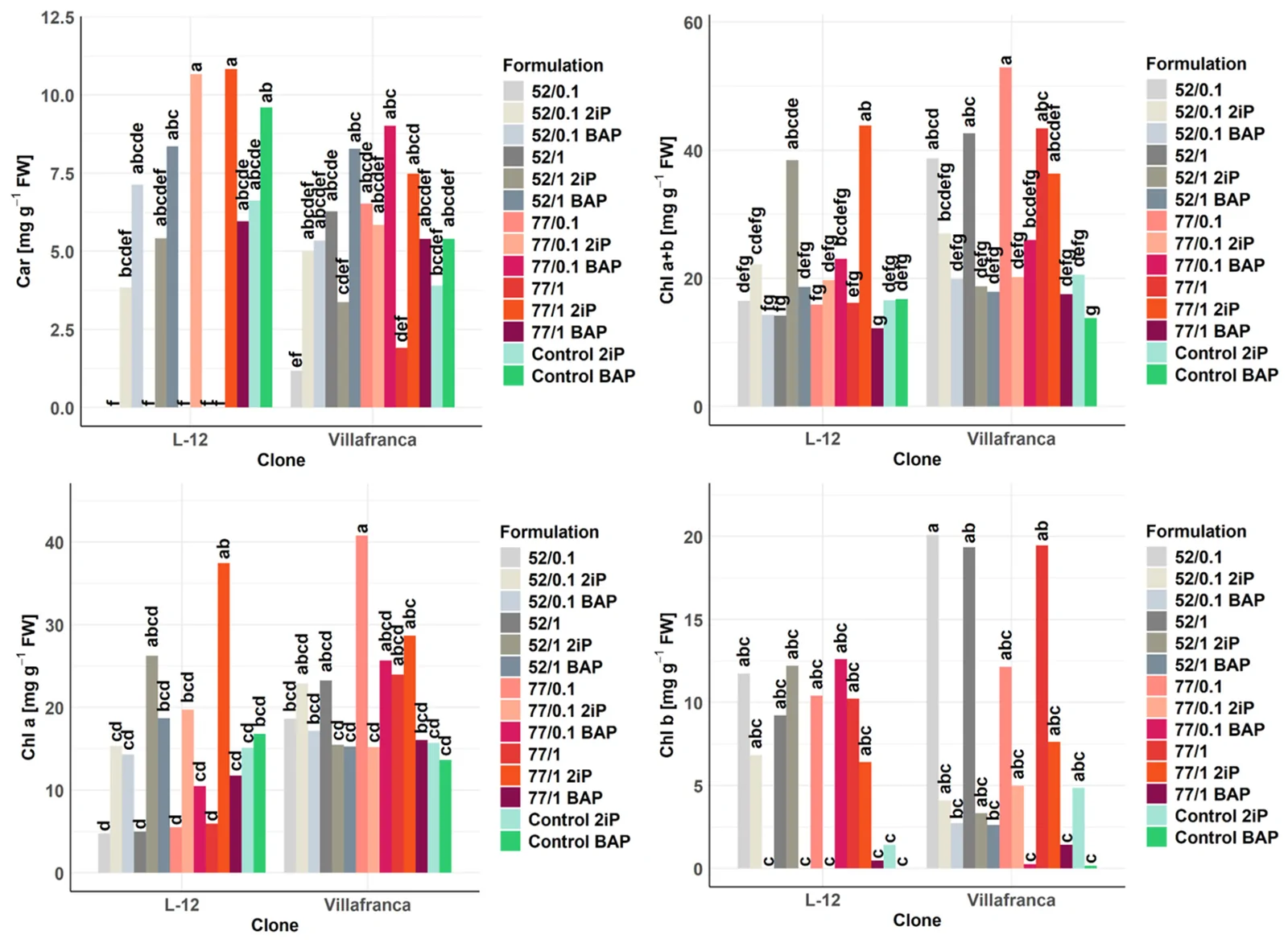
Results of Duncan’s test for photosynthetic pigment content in white poplar clones (L-12 and Villafranca) after 48 days in vitro. Car—carotenoids; Chl a—chlorophyll a; Chl b—chlorophyll b; Chl a+b—total chlorophyll content (all expressed as mg g^−1^ FW). Different lowercase letters above bars indicate statistically significant differences among clone × formulation combinations (Duncan’s multiple range test, *p* ≤ 0.05). Treatment labels: The first part of the label represents the CKX inhibitor, the second its medium concentration in µM, and the third part, 2iP and BAP in medium at a concentration of 1 µM. Control media contained no CKX inhibitor.

An all-or-nothing pattern for carotenoids in clone L-12: several formulations had near-zero values, whereas, just one column away, 77/0.1 2iP and 77/1 yield the highest values on the entire graph (~10.7–10.9); for Villafranca, no zero values appear, with the highest value observed for 77/0.1 BAP (~9.0) (Figure 4). Total chlorophyll (Chl a+b) is highest for Villafranca at 77/0.1 (~53), clearly exceeding the other columns. For chlorophyll a, the clone × formulation interaction was significant (*p* = 0.041), although the main effect of the formulation was not significant (*p* = 0.165; Table 1). The highest value was recorded for Villafranca with 77/0.1 (~41), close to L-12 with 77/1 2iP (~37.5), while several L-12 formulations remained at the lowest values (~5–6). For chlorophyll b, the clone x formulation interaction was not significant (*p* = 0.628), and most combinations did not differ significantly from one another. The three highest values belong to Villafranca (52/0.1, 52/1, 77/1; ~19–20), whereas several formulations across both clones show values close to zero.

There were differences between clones in their reaction to the examined treatments of formulation. The highest multiplication rate, significantly higher than on Control BAP and Control 2iP, was found in Villafranca on 77/1 and 52/0.1 (4.22 and 5.57, respectively), but in L-12 differences between formulations were in most cases non-significant. Villafranca also achieved a high multiplication percentage (over 95%) on 52/0.1, 77/1, 77/1 2iP and 52/1, but in L-12 the highest MP, found on BAP, achieved just above 70%. The highest shoot fresh weight per explant in L-12 was found on 77/1 2iP (0.458 g/plant), while Villafranca achieved 0.566 g/plant on 77/1. The differences in dry mass between formulations were not significant in L-12, while in Villafranca the highest values were observed for 77/1 and 77/0.1. For clone L-12, the highest values for chlorophyll a, carotenoid, and total chlorophyll (a+b) content were recorded with the 77/1 2iP treatment, while the highest numerical values for chlorophyll b were observed with 52/1 2iP and 77/0.1 BAP (exceeding 12 mg/g FW), with no significant differences compared to most other formulations. On average, the Villafranca clone exhibited a significantly higher content of chlorophyll a and total chlorophyll, whereas no significant difference between the clones was found for chlorophyll b and carotenoids. In the Villafranca clone, the highest values for chlorophyll a and total chlorophyll (52.94 mg/g FW) were obtained with the 77/0.1 treatment; for chlorophyll b, with 52/0.1 and 77/1 (approximately 20 mg/g FW); and for carotenoids, with 77/0.1 BAP and 52/1 BAP (exceeding 8 mg/g FW) Table S1.

### 2.2. Biochemical Parameters

**Figure 5:**
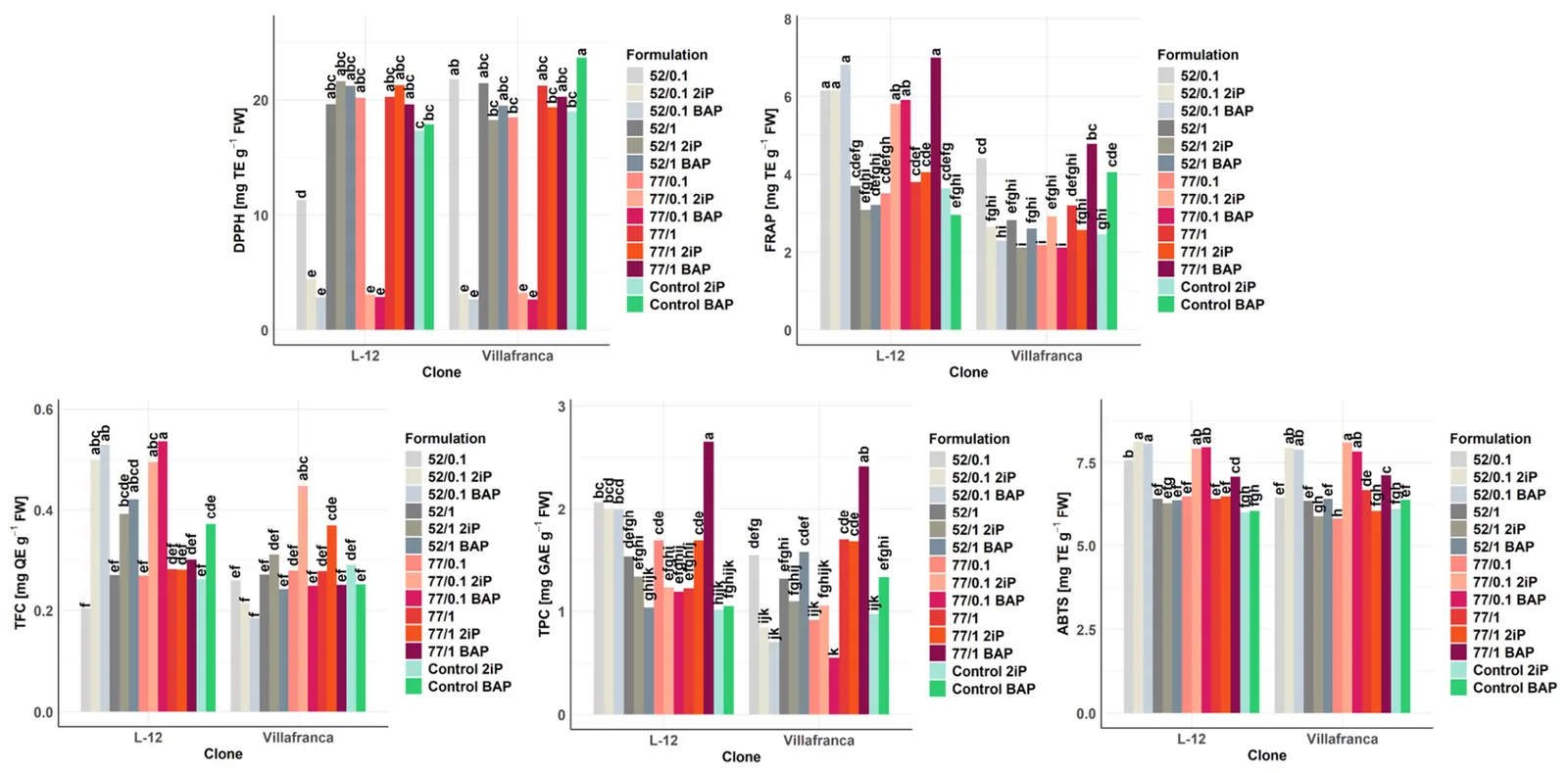
Results of Duncan’s test for phenolic compound content and antioxidant capacity in white poplar clones (L-12 and Villafranca) after 48 days in vitro. DPPH—DPPH radical scavenging activity; ABTS—ABTS radical scavenging activity; FRAP—ferric-reducing antioxidant power; TPC—total phenolic content; TFC—total flavonoid content. Different lowercase letters above bars indicate statistically significant differences among clone × formulation combinations (Duncan’s multiple range test, *p* ≤ 0.05). Treatment labels: The first part of the label represents the CKX inhibitor, the second its medium concentration in µM, and the third part, 2iP and BAP in medium at a concentration of 1 µM. Control media contained no CKX inhibitor.

The phenolic compound content and antioxidant capacity parameters were analyzed using the same statistical procedure, and the results of the Duncan test are presented in Figure 5 and Table S2. The total phenolic content, total flavonoid content, DPPH and ABTS radical scavenging capacity, and ferric-reducing antioxidant power were determined. As with the morphometric parameters, the values are presented separately for the clone effect, the formulation effect, and their interaction, making it possible to observe whether a specific formulation acts in the same way on both clones or only on one of them.

Clone L-12 exhibited higher values than Villafranca regarding phenolic compound content, flavonoids, and ABTS activity. The most significant difference was observed in the FRAP assay, 4.904 versus 2.946, where the clone effect was the strongest across the entire analysis (*F* = 140.996). Regarding DPPH, the difference between the clones was not statistically significant (*p* = 0.097).

Medium formulation was the most influential factor for DPPH and ABTS, which also showed the highest F-test values. The experiment’s pattern is most clearly visible here: the four variants involving the application of a 0.1 µM CKX inhibitor alongside a cytokinin stood out distinctly from the rest; DPPH values dropped below 4, whereas they remained above 15 on the other media. These same four variants also yielded the highest ABTS values, all falling within the same significance group. Flavonoid levels followed a similar trend, peaking with the 77/0.1 2iP treatment. However, for phenolics and FRAP, the maximum values were obtained at the higher concentration, specifically with the 77/1 BAP medium. The highest DPPH values were recorded on the control media, in the absence of inhibitors.

There is a distinct cluster of high DPPH values ranging from 18 to 23.5, with the highest observed in Villafranca on the BAP control, separated from a group of low values (2.5–3.2) associated with formulations containing BAP or 2iP at a concentration of 0.1 μM for both clones (Figure 5). FRAP results reveal a consistent difference between the clones: the highest values are consistently found in L-12 (maximum of ~7.0 with 77/1 BAP), while the lowest occur in Villafranca (minimum of ~2.1 with 77/0.1 BAP). Flavonoid content (TFC) peaks in L-12 on the 77/0.1 BAP medium (~0.54) and is also lowest in L-12 on the 52/0.1 medium (~0.20); notably, this same formulation does not yield the lowest value for Villafranca. Total phenolic content (TPC) reaches its maximum with the 77/1 BAP formulation for both clones (L-12: ~2.67; Villafranca: ~2.42), while the absolute minimum is observed in Villafranca on the 77/0.1 BAP medium (~0.55). ABTS activity exhibits the least variation among all biochemical parameters, with values ranging from ~5.8 to ~8.1.

The interaction between clone and formulation was significant for all five parameters, indicating that the same medium did not elicit the same response from both clones. One treatment stood out consistently: 77/1 BAP yielded the highest phenolic content for both Villafranca and L-12. Regarding FRAP, there was a substantial gap: L-12 reached 7.003, while Villafranca topped out at 4.789 on the same medium. The same pattern was observed for flavonoids. However, a decrease in DPPH activity in the variants treated with 0.1 µM CKX inhibitor combined with a cytokinin was observed in both clones.

### 2.3. Analysis of Variance for the Examined Parameter

The significance of the sources of variation was tested using two-way analysis of variance, with clone and medium formulation as fixed factors. F-test values and associated probabilities are presented in Table 1 for both main effects and their interaction, covering all 18 measured parameters, including the rooting parameter. Table 1 indicates which parameters were significantly influenced by genotype, which by culture medium composition, and for which the response to treatment depended on the clone.

The F-test was significant in all traits in all sources of variation except for the effect of clone on Chl b, Car, and DPPH, the effect of formulation on Chl a, and the effect of their interaction on MP and Chl b.

The F-test outcomes for the two-way factorial analysis revealed that each of the morphometric and biochemical indicators under consideration, encompassing shoot height (SH), multiplication rate (MR), moisture content (MC), shoot fresh weight (SFW), shoot dry weight (SDW), chlorophyll a content (chl a), chlorophyll b content (chl b), carotenoids content (Car), chlorophyll a and b content (chl a+b), total phenolic content (TPC), Total flavonoids content (TFC), DPPH radical scavenging capacity (DPPH), ABTS radical scavenging capacity (ABTS), ferric-reducing antioxidant power (FRAP), were overall significantly influenced by the clone type, the specific formulation used, and their interaction. The data showed that the impact of clone was an exception for Chl b, Car, and DPPH, while the effect of formulation was an exception for Chl a, and the combined effect of clone X formulation also presented exceptions for MP and Chl b.

**Figure 6:**
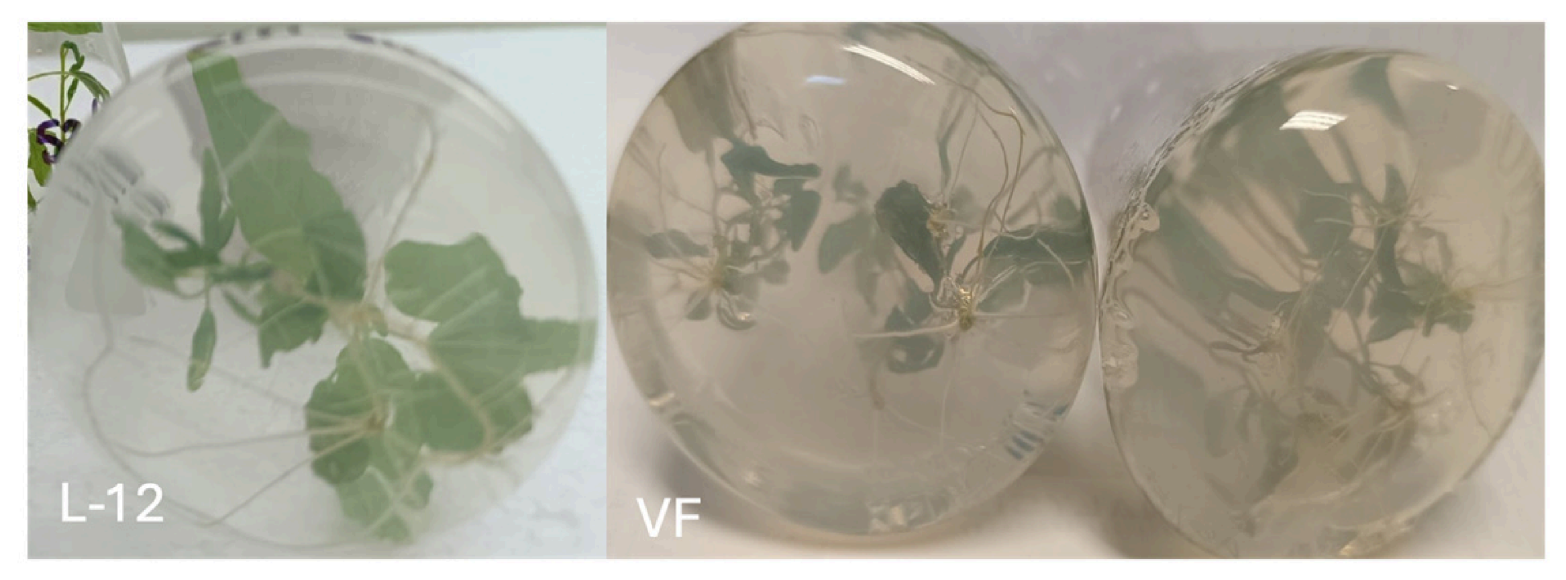
Root system of explants of clones L-12 (**left**) and Villafranca (VF; **right**) of *Populus alba* after 48 days of culture on MS medium without exogenous cytokinins, supplemented only with compound **52** (0.1 µM). Clone Villafranca formed a well-developed root system, while clone L-12 had sporadic rooting, with few but longer roots.

**Figure 7:**
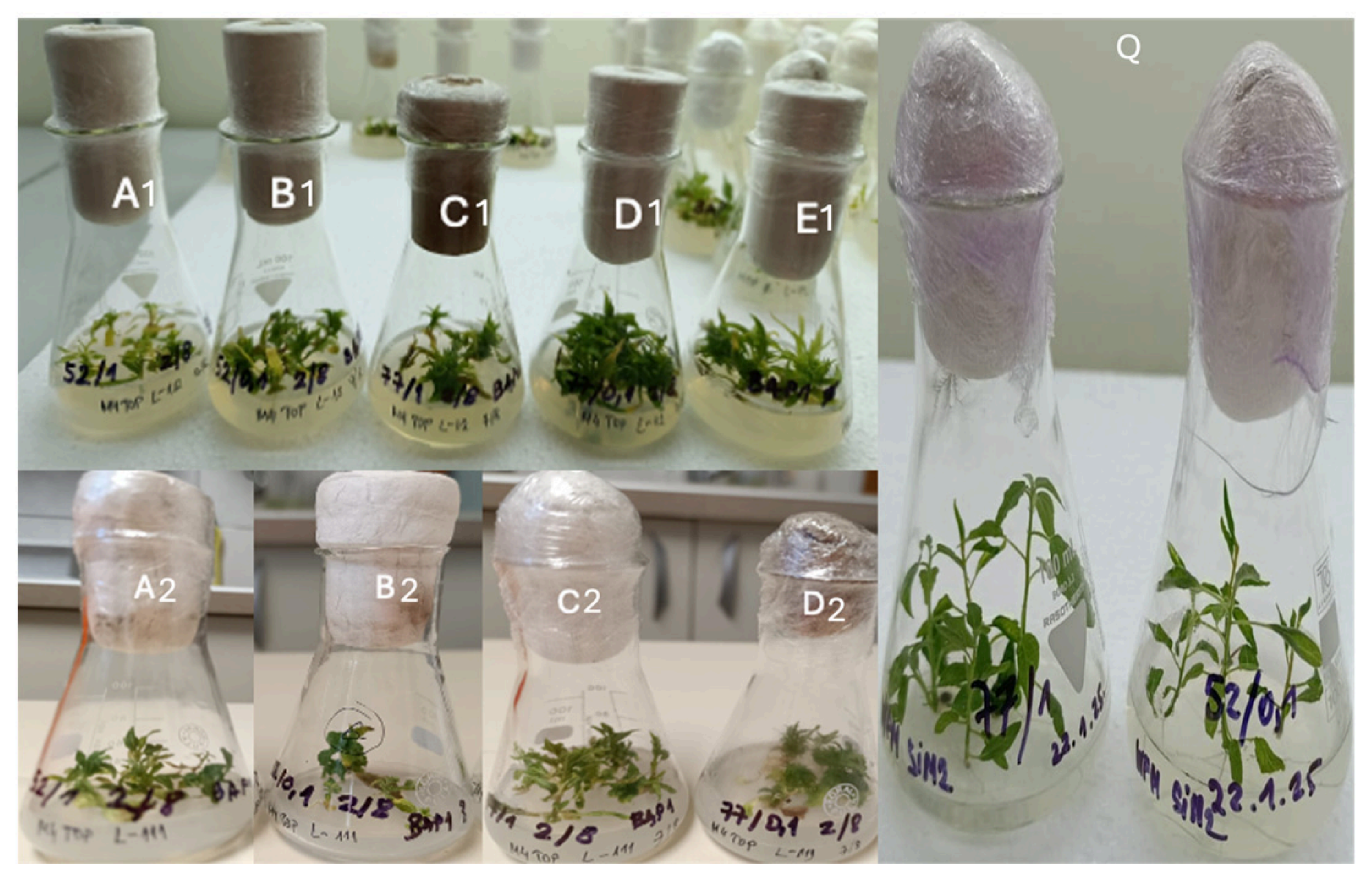
Explants of the L-12 and Villafranca clones of *Populus alba* after 48 days of culture on MS medium. Top row—clone L-12 on medium containing BAP (1 µM): A1—compound **52**, 1 µM; B1—compound **52**, 0.1 µM; C1—compound **77**, 1 µM; D1—compound **77**, 0.1 µM; E1—control (BAP 1 µM, without CKX inhibitor). Bottom row—clone Villafranca on medium containing BAP (1 µM): A2—compound **52**, 1 µM; B2—compound **52**, 0.1 µM; C2—compound **77**, 1 µM; D2—compound **77**, 0.1 µM. Q—clone L-12 on medium without exogenous cytokinins, supplemented only with a CKX inhibitor: compound **77**, 1 µM (**left**) and compound **52**, 0.1 µM (**right**).

**Figure 8:**
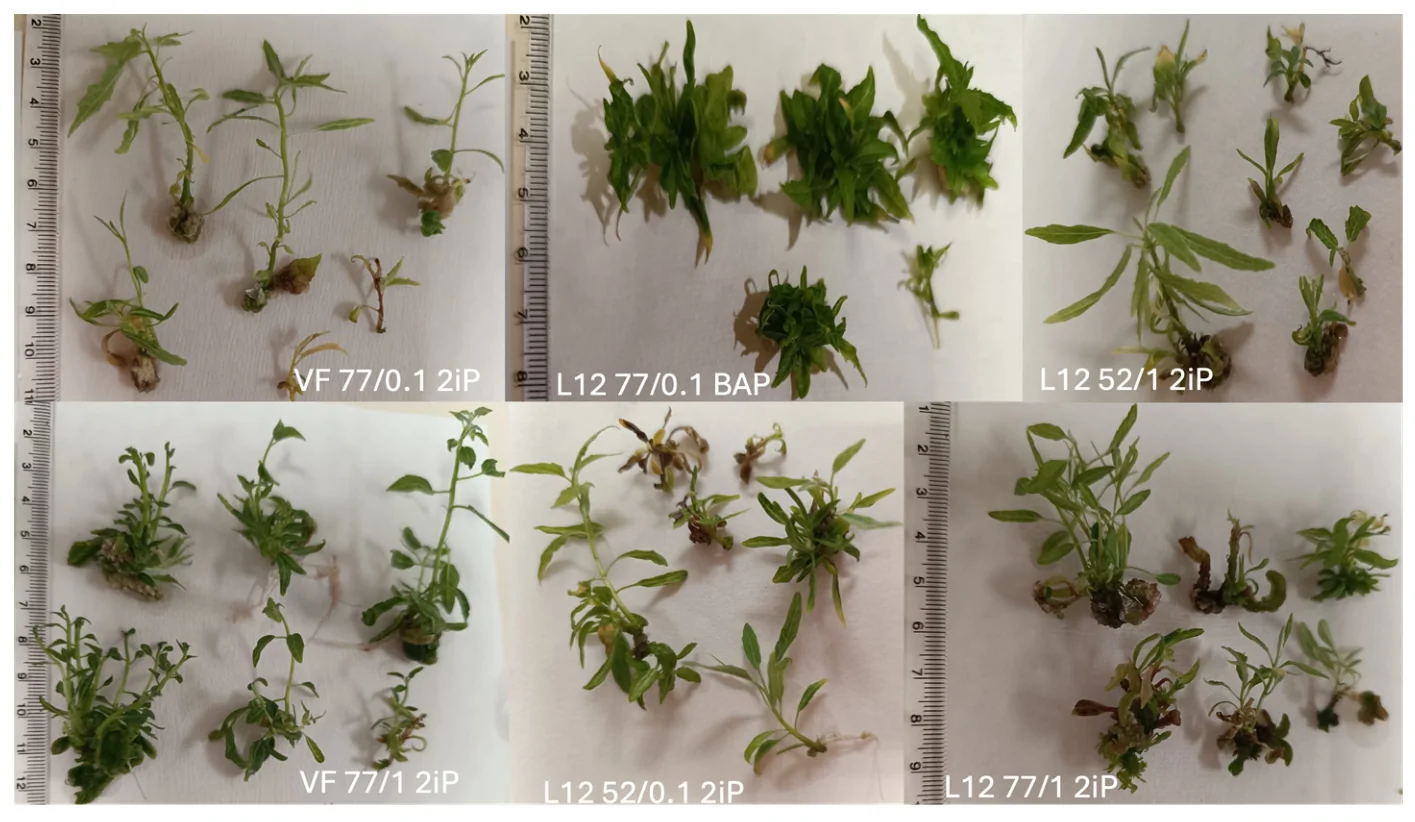
Representative explants of *Populus alba* clones Villafranca (VF) and L-12 after 48 days of culture on MS medium containing CKX inhibitors in combination with an exogenous cytokinin (2iP or BAP, 1 µM). (**Top row**): Villafranca, compound **77** (0.1 µM) + 2iP; L-12, compound **77** (0.1 µM) + BAP; L-12, compound **52** (1 µM) + 2iP. (**Bottom row**): Villafranca, compound **77** (1 µM) + 2iP; L-12, compound **52** (0.1 µM) + 2iP; L-12, compound **77** (1 µM) + 2iP.

**Figure 9:**
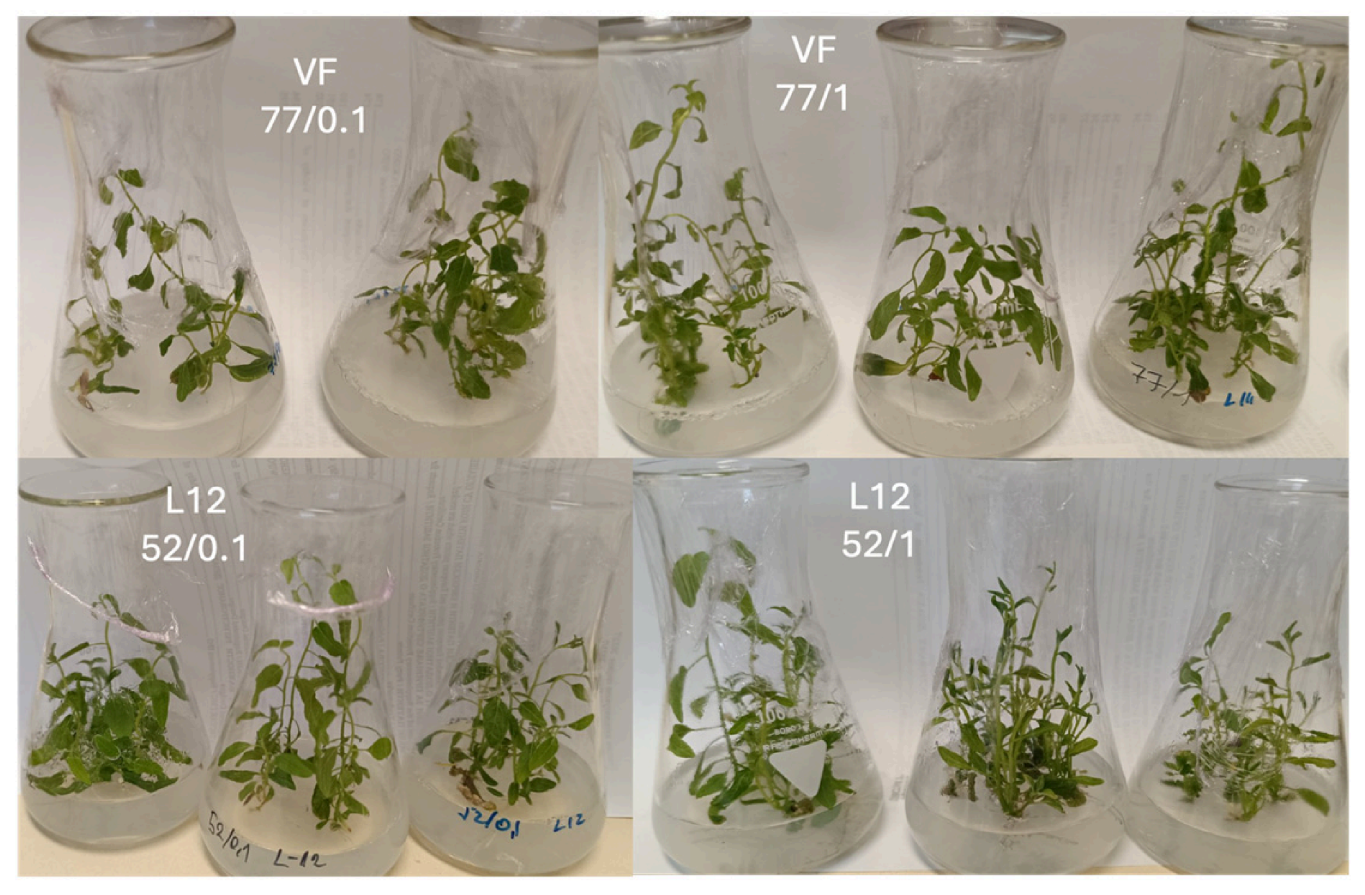
Explant cultures of *Populus alba* clones Villafranca (VF) and L-12 after 48 days of growth on MS medium lacking exogenous cytokinins, supplemented only with a CKX inhibitor. (**Top row**): Villafranca, compound **77** at 0.1 µM (left) and 1 µM (right). (**Bottom row**): L-12, compound **52** at 0.1 µM (left) and 1 µM (right).

Rooting occurred almost exclusively on media containing only the CKX inhibitor. On these media, the Villafranca clone formed a well-developed root system, whereas rooting was sporadic for L-12 (Figure 6). Explants of the L-12 and Villafranca clones of *Populus alba* after 48 days of culture on MS medium are provided in Figure 7, Figure 8 and Figure 9.

### 2.4. Principal Component Analysis

**Figure 10:**
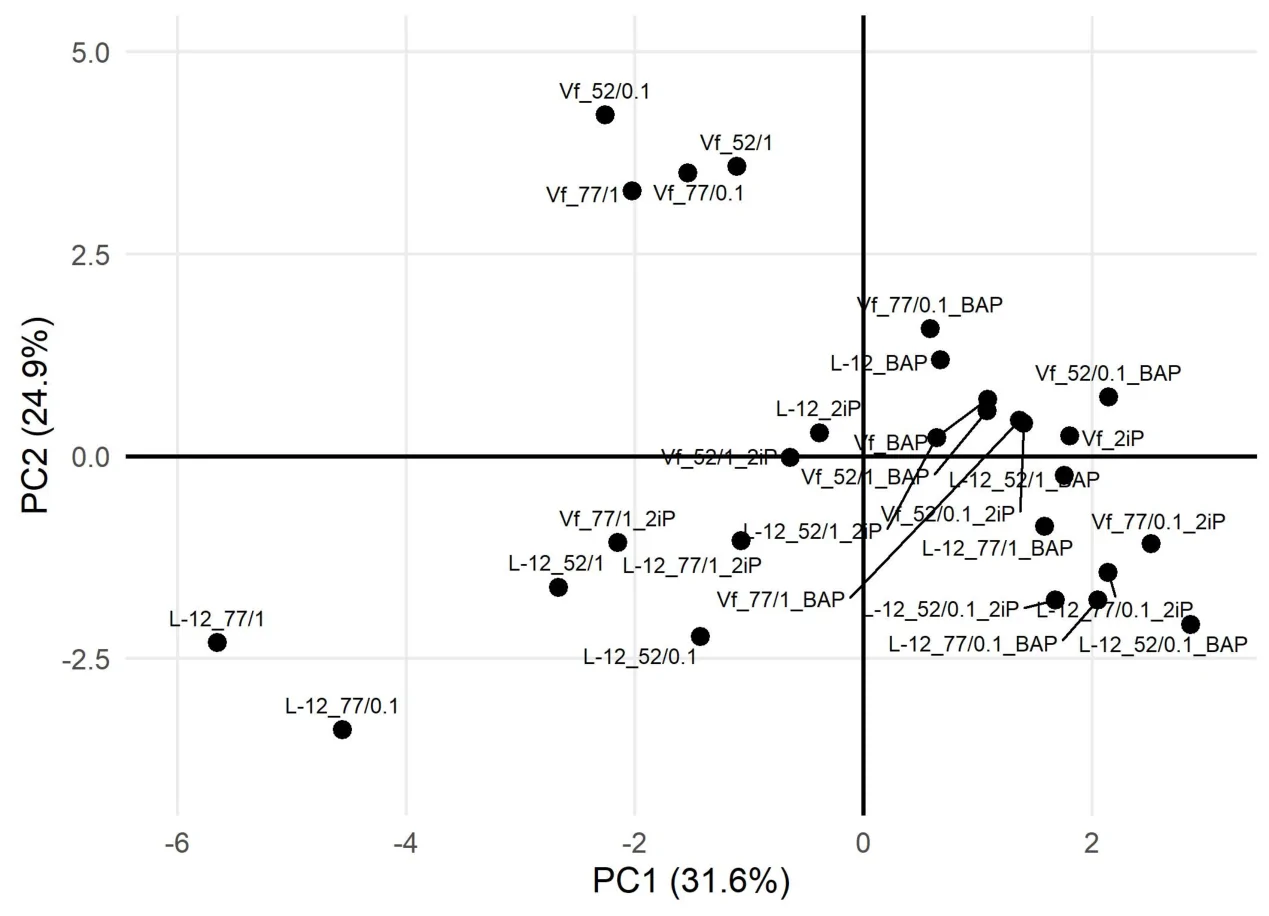
The relationship among treatments of the interaction Clone × formulation according to the scores of the first two unrotated principal components. Vf stands for clone Villafranca; Treatment labels are as in Figure 2.

The relationships between the analyzed characteristics and the applied treatments were examined using principal component analysis at two levels. Rooting parameters were not included because rooting occurred only on four formulation treatments. The first level concerns the characteristics: their loadings on the first five principal components, rotated using the Varimax method, are presented in Table 2 and allow for the identification of parameter groups that vary together. The second level concerns the treatments and is illustrated in Figure 10, which shows the position of each clone-formulation combination relative to the scores of the first two unrotated components. Two representations are complementary: the first shows which parameters are interrelated, while the second indicates which treatments are similar in terms of explant response.

The relationship between characteristics is described according to their loadings with the first five principal components, rotated by Varimax rotation. These five principal components describe 88.5% of the total variance. The first five principal components were selected to be rotated because they met Kaiser’s rule, i.e., their eigenvalues were higher than 1. Thus, the original variables were divided into five groups: the first, which included MC, SFW, SDW, Chl a, Chl b, and Chl a+b; the second, including SH, MR, MP; the third, with DPPH, ABTS, and TFC; the fourth, with Car; and the fifth, with TPC and FRAP.

The first two principal components describe 56.5% of the total variance, whose scores are presented in Figure 10. Formulation treatments in Villafranca and L-12 are separated along the second principal component. Also, most of the treatments with a concentration of 0.1 µM 52 or 0.1 µM 77 are on the positive side of PC1, and most of the media with 1 µM 52 or 1 µM 77 are on the negative side of PC1. Also, most of the treatments with no **52** or **77** compound are on the positive side of PC1, while all the treatments with no 2iP or BAP are on the negative side of the same principal component (Figure 10). Culture media lacking exogenous cytokinin and supplemented only with a CKX inhibitor are separated from all other treatments along PC1 (negative scores). These were the only media where rooting occurred; since the rooting percentage was not included in the PCA, this association is indirect.

According to the principal components analysis (PCA) of the relationship among treatments of the interaction clone × formulation, a few groups of treatments differentiate the most from the other treatments. The first group is 77/1 µM and 77/0.1 µM on clone L-12; the second group contains the treatments 52/1 µM, 52/0.1 µM, 77/1 µM, and 77/0.1 µM. The main cluster of treatments is most different from these two groups of treatments. From the cluster of treatments, some treatments show similar results. When considering clone L-12, the effects observed from treatments at 52/1 µM, 52/0.1 µM, and 77/1 µM 2iP are similar to each other, and this similarity also extends to the 77/1 µM 2iP treatment on clone Villafranca. The treatments involving 52/0.1 µM 2iP, 52/0.1 µM BAP, 77/0.1 µM 2iP, and 77/0.1 µM BAP all demonstrated comparable outcomes. These alternative treatments are categorized collectively within a more substantial cluster, illustrating consistent patterns while also acknowledging the diversity present among them.

## 3. Discussion

This study evaluated the effect of two cytokinin oxidase/dehydrogenase inhibitors, compounds **52** and **77**, on the in vitro micropropagation of two *Populus alba* clones. Both compounds influenced the response of the explants, but the direction and magnitude of the response varied depending on the compound, the applied concentration, and the clone. Besides significant differences between examined clones for most parameters except Chl b and Car, there were also significant differences in their reaction to the presence and concentrations of examined active substances in the examined media formulations. This genotype-dependent variability is consistent with previous observations regarding these inhibitors, which have elicited favorable responses in some species and negligible ones in others [20,21].

It should be emphasized that neither CKX enzymatic activity nor endogenous cytokinin levels in the explants were determined in the present study. Therefore, the link between the observed responses and CKX inhibition is indirect: it relies on the inhibitory activity previously demonstrated for compounds **52** and **77** [20,21] and on the similarity between the phenotypes obtained and those associated in the literature with an increased availability of cytokinins. Off-target or non-specific effects of the compounds cannot be ruled out. Diphenylurea derivatives may also influence morphogenetic processes through other mechanisms; for instance, some stimulate adventitious rhizogenesis [22], and a certain degree of toxicity has been reported for compound **52** [21]. Therefore, the responses described below cannot be attributed solely to CKX inhibition, and the mechanistic interpretations provided in this section should be viewed as hypotheses consistent with the obtained data, which require confirmation through the determination of CKX activity and the endogenous cytokinin profile.

Of the two CKX inhibitors examined, compound **77** yielded the best overall results regarding the measured parameters. Maximum shoot height values were recorded on media 77/1 and 77/0.1, while the highest multiplication rate was achieved on 77/1, significantly exceeding that of the 2iP control. The favorable effect of CKX inhibitors on multiplication and regeneration in tissue culture has also been reported by other authors; for instance, in *Lobelia*, *Drosera*, and *Plectranthus*, diphenylurea-type compounds led to significant increases in shoot regeneration frequency [20,21,23]. A result more similar to the case analyzed here comes from *Melia volkensii*, a woody species in which a CKX inhibitor reactivated meristematic zones that were inactive under standard culture conditions [24], which suggests that these compounds may also be active in woody species. Unlike the study on *Melia volkensii*, where a purine-type inhibitor was applied to leaf explants at 10 µM and rooting was not evaluated, in the present study, diphenylurea-type inhibitors were associated at much lower concentrations, with both multiplication and rooting. These results suggest that the tested CKX inhibitors may be useful in optimizing the multiplication medium for white poplar clones.

On media containing only inhibitors (**52** or **77**), most morphological parameters showed higher values than on the control media supplemented with 2iP or BAP. Moreover, the absence of exogenous cytokinins even allowed the formation of a root system. Differences from controls, including rooting, cannot be attributed solely to the effect of CKX inhibitors and should be examined in further studies.

The formation of the root system was specific to these media and extremely rare in any of the treatments that included the examined exogenous cytokinins. The stimulatory effect of diphenylurea derivative 2,3 MDPU on adventitious rooting was reported by Ricci et al. [22], who found that it can improve the effect of indole-3-butyric acid in rooting stimulation in apple one-nod explants in vitro. However, for the Villafranca clone, the number of roots was significantly lower at 52/1 than at 52/0.1, whereas for compound **77**, the difference between the two concentrations was not significant (Figure 3). This result suggests that caution should be exercised when using compound **52** at higher concentrations for the purpose of inducing adventitious root formation [25].

The better performance of CKX inhibitor-only media was evident in photosynthetic pigment content. While the content of chlorophyll a varied poorly between formulation treatments, the content of chlorophyll b and Chl a+b was significantly higher on these media compared to control media. The higher pigment content may reflect a better physiological state of the explants on these media in clone Villafranca.

The effect of compound **77** did not follow a linear relationship with concentration. Although the 1 µM 77 variant yielded the best values for shoot height and multiplication rate, the 0.1 µM concentration was among the variants with the highest values for other parameters, including total flavonoid content (77/0.1 2iP) and antioxidant capacity as determined by the ABTS method. On the other hand, CKX inhibitor-only media and control media with either 2iP or BAP showed lower values of TPC and TFC than media that included both inhibitors and exogenous cytokinin. The same pattern was found for ABTS and FRAP, but the opposite for DPPH. This discrepancy can be partially explained by the differences between the two methods: the DPPH radical reacts more slowly or incompletely with certain phenolic compounds, whereas ABTS reacts with both hydrophilic and lipophilic antioxidants [26,27]. An increase in phenolic compound content is frequently associated with a stress response [28], which may indicate that the combination of the CKX inhibitor and an exogenous cytokinin more strongly activated the explants’ antioxidant system. Confirming this hypothesis would require direct measurements of reactive oxygen species or oxidative degradation. The optimal concentration therefore depends on the specific parameter being monitored, an observation also supported by principal component analysis, in which treatments at 0.1 µM separated from those at 1 µM along the first principal component. The need for fine-tuning the dosage is also discussed in the literature on these compounds, where selectivity and efficacy are analyzed in relation to concentration and the context of application [20,21].

Compound **52** exhibited a different action profile. The maximum effect was achieved at the lower concentration of 0.1 µM, particularly in the Villafranca clone, where it yielded the highest multiplication rate in the experiment. For clone L-12, the same treatments did not yield significant differences compared to the control, and the maximum height for this clone was also achieved with compound **52** at 0.1 µM (6.9 cm). Thus, the two compounds are not interchangeable; each exhibited a distinct action profile, determined by the genotype. This behavior suggests the existence of an optimal dosage window: if CKX inhibition leads to an increase in endogenous cytokinin levels, excessive levels could inhibit elongation and especially adventitious root formation [12,19], which corresponds to the decrease in root number observed at higher concentrations. An alternative explanation, which cannot be ruled out, involves non-specific effects of the compound at higher concentrations, given that a certain degree of toxicity has been reported for compound **52** [21]. To confirm these hypotheses, it is essential to test a more extensive range of concentrations.

Differences between the two clones were observed across most analyzed parameters, though not in the same direction. The Villafranca clone exhibited higher values for the multiplication rate and percentage, shoot biomass, and both chlorophyll a and total chlorophyll content, whereas the L-12 clone produced taller shoots and accumulated greater quantities of phenolic compounds and flavonoids, along with a higher antioxidant capacity (ABTS, FRAP). Consequently, shoot elongation was not directly correlated with multiplication or biomass accumulation: the two clones allocated their resources differently, with L-12 favoring elongation, while Villafranca favored branching and biomass accumulation. The separation of the clones along the second principal component supports this interpretation. This divergence may reflect different adaptive strategies of the two clones, also observed in an in vitro drought study, in which L-12 responded with a more pronounced increase in antioxidant capacity and osmoprotectant content compared to Villafranca [15]. A genotype-dependent response to the same hormonal regime has also been reported in other micropropagated woody species, with authors concluding that the protocol requires separate optimization for each genotype [29].

The maximum values for chlorophyll a, carotenoids, and total chlorophyll (a+b) observed with the 77/1 2iP treatment in clone L-12 can be linked to the well-known role of cytokinins in chloroplast development and function. Cytokinins stimulate chlorophyll biosynthesis and delay the degradation of the photosynthetic apparatus [30]; thus, maintaining higher levels of active cytokinin by inhibiting CKX would theoretically support this effect. On the other hand, the data obtained in Villafranca point to the 0.1 µM concentration of compound **77** alone, which resulted in a trend of increased total and chlorophyll a content. However, the interpretation remains cautious, as the analysis of variance showed no significant effect of the formulation factor on chlorophyll a (Chl a) content. In apple, it has been demonstrated that the type of cytokinin in the culture medium alters both chloroplast ultrastructure and pigment content, with the response being cultivar-dependent [29], an observation consistent with the differences noted here between Villafranca and L-12.

The content of phenolic compounds and the antioxidant capacity parameters showed significantly higher values in clone L-12 across all measurements, except for DPPH. The highest DPPH value was recorded on the control medium with BAP, i.e., in the absence of inhibitors, which suggests that the presence of inhibitors did not increase the antioxidant capacity measured by this method. In a study on white poplar clones, including Villafranca, antioxidant capacity and osmoprotectant content served as indicators of the stress level experienced by explants [8,15], and the same five parameters (TPC, TFC, DPPH, ABTS, and FRAP) were applied to poplar and willow clones grown under pollution conditions [31]. The relationship between CKX activity and the antioxidant system has been directly demonstrated in cotton, where the modulation of a CKX gene triggered an antioxidant response under salinity stress [11]. However, these parameters are indirect indicators, and assessing oxidative stress would require direct measurements.

A result of practical relevance is the favorable effect on rooting observed on media lacking cytokinins. Exogenous cytokinins inhibit adventitious root formation by suppressing the initiation of root primordia [19], and in poplar, in vitro rooting remains a sensitive stage that has been the subject of specific studies [17]. The formation of a well-developed root system in Villafranca clone explants on media containing only inhibitors and no exogenous cytokinins indicates that these compounds can be used without compromising in vitro rooting, at least for this clone. This result could allow for a simplified protocol, as the same medium supports multiplication without inhibiting rooting; however, practical applicability depends on plant behavior during acclimatization, which was not evaluated in this study. This observation is consistent with the results of the principal component analysis, in which treatments lacking exogenous cytokinins were associated with explant growth and multiplication.

The low number of roots found in clone L-12 suggests that **52** and **77** compounds induced stronger inhibition of root initiation in comparison to Villafranca. This indicates a clear genotype-specific response of the examined clones. Considerable differences in the reaction of the white poplar clones were also demonstrated in the study of low pH tolerance in vitro [32]. One possible explanation of this inhibitory effect, which was not experimentally verified in the present study, is that CKX inhibition might have led to the accumulation of endogenous cytokinins, which, in the absence of exogenous auxin, might have inhibited root initiation [12,19]. Other explanations cannot be ruled out, including non-specific effects of the compounds or differences between clones regarding their uptake and metabolism.

By the end of the 48 days, the explants showed no signs of vitrification or senescence. The absence of hyperhydricity is significant, as this physiological disorder is one of the most common issues in in vitro culture and is associated, among other factors, with hormonal imbalance in the medium [33]. The absence of senescence aligns with the role of cytokinins in delaying leaf aging, a role also demonstrated in studies on CKX genes, where reduced enzyme activity delayed the onset of senescence [13].

The results indicate that, for the two clones tested, CKX inhibitors can be integrated into the micropropagation protocol for *Populus alba*, provided that the choice of compound and concentration is tailored to the specific clone and the intended objective. Compound **77** at 1 µM yielded the best results for biomass accumulation, whereas compound **52** at 0.1 µM yielded superior results for the Villafranca clone regarding multiplication and shoot elongation in the L-12 clone. The study’s limitations stem from the small number of clones tested and the fact that the evaluation was confined to the in vitro phase. CKX activity and endogenous cytokinin levels were not measured in this study. The observed effects are consistent with the inhibitory activity against CKX previously demonstrated for these compounds [20,21]; however, confirming the mechanism requires direct measurements. Plant behavior during acclimatization and under field conditions remains to be verified, particularly given that the activity of these enzymes also influences whole-plant architecture [7].

## 4. Materials and Methods

### 4.1. Plant Material and Experimental Design

In vitro cultures were initiated from adventitious buds collected in spring from juvenile side branches of two *Populus alba* clones, L-12 and Villafranca. Both clones were chosen by positive selection for straight trunk, vigorous growth, and tolerance to prevalent biotic and abiotic challenges. The material originated from the gene pool of the experimental estate Kać (N 45°17′, E 19°53′, 78 m. a. s. l.) of the Institute of Lowland Forestry and Environment, University of Novi Sad, Serbia. The buds were washed under running tap water with a few drops of detergent for 30 min, then immersed in 70% ethanol for 30 s and surface-sterilized in a 0.1% mercuric chloride (HgCl_2_) solution for 7 min, the buds were rinsed three times with sterile distilled water and placed in Erlenmeyer flasks containing solid basal MS medium [34] supplemented with the hormones BAP (1.0 mg/L^−1^), IBA (0.1 mg/L^−1^) and GA3 (0.1 mg/L^−1^) as the initiation medium. After the initiation step, micropropagation was performed on solid MS basal medium [34] with added 8 g L^−1^ agar and 30 g L^−1^ sucrose. The pH of the medium was adjusted to pH 5.7 with 1 N HCl or 1N NaOH before sterilization. Apical segments from elongated shoots were transplanted to MS medium fortified with 2iP (1 µM) and BAP (1 µM). This procedure was used for the experiment with CKX inhibitors, with the objective of evaluating their effect on the micropropagation efficiency of poplar clones. For the cytokinin oxidase/dehydrogenase (CKX) inhibitors in this experiment, compounds **52** (1-(3,5-dichlorophenyl)-3-(3-(trifluoromethoxy)phenyl)urea) and **77** (2-(3-(3,5-dichlorophenyl)ureido)-4-methoxybenzamide) were used. The compounds were dissolved in DMSO as 10 mM stock solutions and added to the culture medium to final concentrations of 1 µM and 0.1 µM; the final DMSO concentration in the medium did not exceed 0.01% (*v*/*v*), applied alone or combined with the cytokinins 2iP (1 µM) or BAP (1 µM). The experiment was set up in three replicate jars with 5 explants per jar, with controls that represent MS nutrient medium with 2iP (1 µM) and BAP (1 µM) without CKX compounds. The control media did not contain DMSO. The experimental design did not include a solvent control; although the final concentration of DMSO in the media was very low (≤0.01% *v*/*v*), a potential effect of the solvent on the explants cannot be ruled out. The cultures were grown for 48 days in controlled conditions at 26 ± 2 °C, under a white light of 3500 lx m^−2^ emitted by LED lamps in a 16 h/8 h (day/night) photoperiod.

### 4.2. Morphological, Physiological, and Biochemical Analyses

After 48 days of cultivation, the height of the dominant shoot and the number of new shoots were recorded. In addition, shoot height, multiplication rate (shoots per explant), fresh mass (g plant^−1^), dry mass (g plant^−1^), and moisture content (g H_2_O g^−1^ DW) were recorded from each jar.

The multiplication rate (MR) was calculated as the average number of new shoots formed per explant. The multiplication percentage (MP, %) represented the percentage of explants that formed at least one new shoot. The rooting percentage (RP, %) was calculated as the percentage of explants that formed roots, and shoot height (SH, cm) was measured based on the tallest shoot of each explant.

After the measurement of morphological parameters, the same plant material was used to evaluate the effect of CKX inhibitors on explant growth and development, based on physiological and biochemical parameters. Physiological measurements included parameters related to the content of photosynthetic pigments (mg g^−1^) in the plant tissues, determined by Lichtenthaler & Wellburn [35] for the following pigments: chlorophyll a (Chl a), chlorophyll b (Chl b), and carotenoids (Car), and the following derived parameters: chlorophyll a+b (Chl a+b). In each sample, the following biochemical parameters were measured: total phenolic content (TPC, mg GAE g^−1^, where GAE stands for gallic acid equivalents [36]), total flavonoids content (TFC, mg QE g^−1^, where QE stands for quercetin equivalents [37]), ferric-reducing antioxidant power (FRAP, mg TE g^−1^, where TE stands for Trolox equivalents [38]), radical scavenging activity against ABTS (2,2-Azino-bis(3-ethylbenzthiazoline-6-sulfonic acid), ABTS, expressed in mg TE g^−1^, where TE stands for Trolox equivalents [26]) and DPPH (2,2-diphenyl-1-picrylhydrazyl) radical scavenging activity expressed as mg TE g^−1^, where TE stands for Trolox equivalents [27]. Ethanol extracts were obtained from 100 mg of ground fresh plant material in 2 mL of 96% EtOH and centrifuged in the dark at 4000 RPM for 10 min at room temperature. The supernatant was then used to determine the content of photosynthetic pigments and measure biochemical parameters. All measured biochemical and photosynthetic parameters were determined by the spectrophotometric method on a MultiSkan™ GO spectrophotometer (Thermo Fisher Scientific, Waltham, MA, USA) in triplicate. Physiological and biochemical traits were measured or calculated for all vegetative mass, including leaves and stems.

Biochemical parameters were also determined from the same ethanolic extract, all via spectrophotometry; values are reported based on fresh weight (FW). Total phenolics were determined according to the Folin–Ciocalteu method [36]. The extracts were mixed with deionized water, 20% sodium carbonate, and Folin–Ciocalteu reagent diluted with distilled water in a proportion of 1:2. The absorbance of the reaction mixture was measured after incubation at room temperature for 30 min, at 720 nm. The data were expressed as mg gallic acid equivalent per gram fresh weight (mg GAE/g^−1^ FW). Total flavonoids were estimated according to the method described by Markham [37]. The extracts were mixed with deionized water (1 mL) and aluminum chloride hexahydrate (2.5 mL). After incubation at room temperature for 15 min, the absorbance of the reaction mixture was measured at 430 nm. The data were expressed as mg quercetin equivalents per g fresh weight (mg QE/g^−1^ FW). Free radical scavenging ability was measured using the stable DPPH (2.2-diphenyl-1-picrylhydrazine) radical [27]. Extract (40 μL) was mixed with DPPH reagent (2 mL). The absorbance was measured at 517 nm. The ABTS (2,2′azino-bis (3-ethylbenzothiazoline-6-sulfonic acid)-diammonium salt) assay was based on a method described by Re et al. (1999) [26]. Extract (100 μL) was mixed with ABTS reagent (3 mL). The absorbance was measured at 734 nm. The ferric-reducing antioxidant power (FRAP) assay was carried out according to the procedure described by Benzie and Strain (1999) [38]. Extract (50 μL) was mixed with FRAP reagent (3 mL). The absorbance was measured at 593 nm. The standard curve for antioxidant tests (DPPH, ABTS, and FRAP) was plotted using Trolox solution; the results were expressed as mg Trolox equivalents (TE) per g of the fresh plant material (mg TE/g^−1^ FW).

### 4.3. Statistical Analysis

Pigments and biochemical parameters were determined on a single sample from each jar (three replicates). The experimental unit was the jar: for morphometric parameters, the mean of the five explants within a jar constituted a replicate, resulting in three replicates for each clone × formulation combination (84 observations in total), and all statistical analyses were performed on these means.

The variability of characteristics was analyzed by two-way factorial analysis of variance in a completely randomized design with fixed factors: Clone (two clones) and formulation (14 formulations), using the lm() command in the R programming language. The Type III Sum of Squares was used by the Car package [39]. The significance of differences between treatments was tested by Duncan’s post-hoc test at a significance level of α = 0.05 using the agricolae package [40] in the R programming language. Prior to the statistical analysis, the multiplication rate (MR) was transformed by square-root transformation [$\sqrt{x+1}$], and multiplication percentage (MP) data were transformed by an arcsine transformation to satisfy the assumption of normality.

The relationships among clone × formulation combinations were examined using the scores of the first two unrotated principal components.

Principal component analysis was performed on the mean values of the 28 clone × formulation combinations using the correlation matrix; consequently, all variables were standardized (mean 0, variance 1) prior to analysis. Principal components with eigenvalues greater than 1 (Kaiser’s rule), specifically the first five components, were retained and rotated using the Varimax method. The rooting percentage (RP) was excluded from the analysis due to the high number of zero values, which would have distorted the correlation structure. The loadings of the original variables with the first five principal components rotated by Varimax rotation were used to examine the relationship between the examined characteristics. Because principal components are not correlated with each other, variables that have high loadings on the same principal component have high correlation with each other and low correlation with other variables. Variables that have high loadings on the same principal component were grouped in the same group. Regarding this, Varimax rotation rotates selected principal components in a way that the variance of the loadings of original variables with the same principal component is maximized. The principal component analysis was performed using the psych package in R [41], and the graph presenting scores for the first two principal components was made using the ggplot2 package in the same programming language [42].

## 5. Conclusions

The two tested CKX inhibitors, compounds **52** and **77**, influenced the in vitro micropropagation of the two *Populus alba* clones differently, depending on the compound, concentration, and clone. Compound **77** yielded the best results for shoot growth and biomass accumulation at a concentration of 1 µM, whereas compound **52** was more effective at 0.1 µM, particularly for the Villafranca clone, where it produced the highest multiplication rate in the experiment. Unlike media supplemented with exogenous cytokinins, media containing only CKX inhibitors allowed Villafranca explants to form well-developed roots (with a rooting percentage exceeding 80%), meaning that multiplication and rooting occurred on the same medium. This response was not observed in clone L-12, in which rooting was poor; therefore, multiplication and rooting on a single medium are, under the tested conditions, genotype-specific. The explants exhibited no signs of vitrification or senescence. Consequently, the choice of compound and concentration depends on the specific clone and the parameter being evaluated.

These results suggest that CKX inhibitors can be incorporated into the micropropagation protocol for white poplar clones tested, with optimization specific to each genotype; however, it should be noted that only two clones were tested and the evaluation was limited to the in vitro stage. Furthermore, the experimental design did not include a solvent control (DMSO without inhibitor); although the final concentration of DMSO in the media was very low (≤0.01% *v*/*v*), an effect of the solvent cannot be ruled out. Applicability to other genotypes and woody species, plant performance during acclimatization and ex vitro growth, and the mechanism of action (assessed by measuring CKX activity and endogenous cytokinin levels) remain to be investigated in future studies.

## Figures and Tables

**Table 1:** F-test for the two-way factorial analysis of variance for examined characteristics.

Variable ^(1)^	Clone			Formulation			Clone × Formulation		
	*F*-Value	*p*-Value		*F*-Value	*p*-Value		F-Value	*p*-Value	
SH	20.163	<0.001	** ^(2)^	10.667	<0.001	**	3.503	0.001	**
MR	25.738	<0.001	**	4.971	<0.001	**	5.342	<0.001	**
MP	9.809	0.003	**	2.500	0.010	**	1.614	0.111	
MC	28.048	<0.001	**	7.727	<0.001	**	2.787	0.004	**
RP	36.083	<0.001	**	25.696	<0.001	**	27.000	<0.001	**
RN	92.544	<0.001	**	4.406	0.021	*	6.977	0.004	**
LR	14.053	0.003	**	2.809	0.085		1.786	0.203	
SFW	23.260	<0.001	**	4.403	<0.001	**	2.799	0.004	**
SDW	27.125	<0.001	**	7.345	<0.001	**	4.743	<0.001	**
Chl a	6.449	0.014	*	1.457	0.165		1.977	0.041	*
Chl b	0.699	0.407		2.596	0.007	**	0.830	0.628	
Car	0.484	0.490		4.717	<0.001	**	2.790	0.004	**
Chl a+b	9.764	0.003	**	2.697	0.006	**	2.928	0.003	**
TPC	23.149	<0.001	**	13.894	<0.001	**	6.324	<0.001	**
TFC	27.890	<0.001	**	4.427	<0.001	**	5.182	<0.001	**
DPPH	2.771	0.097		78.599	<0.001	**	4.500	<0.001	**
ABTS	8.518	0.004	**	59.770	<0.001	**	4.197	<0.001	**
FRAP	140.996	<0.001	**	13.024	<0.001	**	7.212	<0.001	**

^(1)^ Labels of the parameters: SH—Height of the highest shoot, MR—Multiplication rate, MP—Multiplication percentage, RP—Rooting percentage, RN—number of roots, LR—length of the longest root, MC—Moisture content, SFW—Shoot fresh weight, SDW—Shoot dry weight, Chl a—Chlorophyll a content, Chl b—Chlorophyll b content, Car—Carotenoid content, Chl a+b—Content of chlorophyll a and b, TPC—Total phenolic content, TFC—Total flavonoids content, DPPH—DPPH radical scavenging capacity, ABTS—ABTS radical scavenging capacity, FRAP—ferric-reducing antioxidant power. ^(2)^ Labels for the probability of F-test: *—*p* < 0.05, **—*p* < 0.01. Analysis of variance for RN and LR was performed only for 52/0.1, 52/1, 77/0.1, and 77/1 formulation treatments.

**Table 2:** Factorial loadings of the first five principal components, rotated by Varimax rotation.

Original Variable ^(1)^	Rotated Principal Component				
	RC1	RC2	RC3	RC4	RC5
SH	0.209	**0.811**	−0.187	−0.368	−0.010
MR	−0.096	**0.964**	−0.117	−0.123	−0.019
MP	0.026	**0.917**	−0.190	−0.049	−0.143
MC	**−0.838**	0.005	0.102	0.423	−0.111
SFW	**0.880**	0.154	−0.083	0.244	0.084
SDW	**0.896**	0.021	−0.109	−0.202	0.029
Chl a	**0.721**	−0.187	−0.005	0.614	0.034
Chl b	**0.679**	0.185	−0.054	−0.599	−0.045
Car	0.016	−0.347	−0.022	**0.873**	−0.021
Chl a+b	**0.933**	−0.041	−0.034	0.140	0.001
TPC	0.094	−0.012	−0.158	0.055	**0.931**
TFC	−0.066	0.007	**0.540**	0.524	0.280
DPPH	0.099	0.216	**−0.940**	−0.028	0.147
ABTS	−0.115	−0.223	**0.889**	−0.028	0.176
FRAP	0.044	−0.165	0.374	−0.001	**0.875**
Eigenvalue	4.223	2.768	2.249	2.229	1.808
Variance proportion of unrotated components in total variance	0.282	0.185	0.150	0.149	0.121
Cumulative variance Proportion	0.282	0.466	0.616	0.765	0.885
Explained variance of rotated components	0.318	0.208	0.169	0.168	0.136
Cumulative explained variance proportion	0.318	0.527	0.696	0.864	1.000

^(1)^ Labels of the parameters: SH—Height of the highest shoot, MR—Multiplication rate, MP—Multiplication percentage, MC—Moisture content, SFW—Shoot fresh weight, SDW—Shoot dry weight, Chl a—Chlorophyll a content, Chlorophyll b—content, Car—Carotenoid content, Chl a+b—Content of chlorophyll a and b, TPC—Total phenolic content, TFC—Total flavonoids content, DPPH—DPPH radical scavenging capacity, ABTS—ABTS radical scavenging capacity, FRAP—ferric-reducing antioxidant power.

## Data Availability

The original contributions presented in this study are included in the article/Supplementary Material.

## Supplementary Materials

The following supporting information can be downloaded at https://www.mdpi.com/article/10.3390/plants15213293/s1, Table S1: Results of Duncan’s test for morphometric and photosynthetic pigment content parameters; Table S2: Results of Duncan’s test for phenolic compound content and antioxidant capacity. Table S3: Results of Duncan’s test for number and length of roots.

## Abbreviations

The following abbreviations are used in this manuscript:

2iP	2-isopentenyladenine
ABTS	ABTS radical scavenging activity
BAP	6-benzylaminopurine
Car	carotenoids
Chl a	chlorophyll a
Chl a+b	total chlorophyll content
Chl b	chlorophyll b
CKX	cytokinin oxidase/dehydrogenase
DMSO	dimethyl sulfoxide
DPPH	DPPH radical scavenging activity
FRAP	ferric-reducing antioxidant power
FW	fresh weight
GAE	gallic acid equivalents
IBA	Indole-3-butyric acid
LR	longest root
MC	moisture content
MP	multiplication percentage
MR	multiplication rate
MS (medium)	Murashige and Skoog medium
PCA	principal components analysis
QE	quercetin equivalents
RN	number of roots
RP	rooting percentage
SDW	shoot dry weight
SFW	shoot fresh weight
SH	shoot height
TE	Trolox equivalents
TFC	total flavonoid content
TPC	total phenolic content
VF (Vf)	Villafranca
