# Supplementary material for: CKX Inhibitors: From Shoot Tip Micropropagation to Rooted Plantlet
Source: Plants (Basel). 2026 Oct 25;15(21):3293. doi: 10.3390/plants15213293 (PMC13765872; doi:10.3390/plants15213293)
Supplement: Supplementary file 1 [file plants-15-03293-s001.zip › Table S1. Results of Duncan’s test for morphometric and photosynthetic pigment content parameters.pdf]

Table S1. Results of Duncan's test for morphometric and photosynthetic pigment content parameters

Clone	Formulation[3]	SH[1]	MR	MP	MC	RP	SFW	SDW	Chl a	Chl b	Car	Chl a+b
Villafranca		2.90 ^{a} [2]	1.59 ^{a}	62.95 ^{a}	0.857 ^{a}	14.757 ^{b}	0.141 ^{b}	0.023 ^{b}	14.81 ^{b}	5.83 ^{a}	4.89 ^{a}	20.63 ^{b}
L-12		2.02 ^{b}	0.68 ^{b}	31.10 ^{b}	0.709 ^{b}	0.025 ^{a}	0.253 ^{a}	0.093 ^{a}	20.40 ^{a}	7.76 ^{a}	5.15 ^{a}	28.16 ^{a}
	52/0.1	4.49 ^{a}	2.24 ^{a}	58.68 ^{abc}	0.663 ^{b}	50.000 ^{a}	0.179 ^{c}	0.071 ^{b}	11.71 ^{b}	15.93 ^{a}	0.59 ^{e}	27.64 ^{abc}
	52/0.1 2iP	1.68 ^{cd}	0.64 ^{cd}	40.22 ^{abc}	0.850 ^{a}	0.490 ^{b}	0.132 ^{c}	0.022 ^{b}	19.13 ^{b}	5.48 ^{abc}	4.43 ^{bcde}	24.60 ^{bc}
	52/0.1 BAP	1.37 ^{d}	0.49 ^{d}	25.72 ^{bc}	0.885 ^{a}	0.000 ^{b}	0.119 ^{c}	0.014 ^{b}	15.45 ^{b}	1.09 ^{c}	6.42 ^{abc}	16.55 ^{bc}
	52/1	3.22 ^{b}	1.73 ^{ab}	78.77 ^{ab}	0.643 ^{b}	41.318 ^{a}	0.193 ^{c}	0.073 ^{b}	14.13 ^{b}	14.29 ^{ab}	3.13 ^{cde}	28.42 ^{abc}
	52/1 2iP	1.66 ^{cd}	0.62 ^{cd}	39.77 ^{abc}	0.856 ^{a}	0.000 ^{b}	0.164 ^{c}	0.024 ^{b}	20.87 ^{ab}	7.77 ^{abc}	4.39 ^{bcde}	28.64 ^{abc}
	52/1 BAP	1.36 ^{d}	0.53 ^{d}	22.02 ^{bc}	0.880 ^{a}	0.000 ^{b}	0.104 ^{c}	0.012 ^{b}	16.97 ^{b}	1.31 ^{c}	8.31 ^{ab}	18.28 ^{bc}
	77/0.1	4.43 ^{a}	1.71 ^{ab}	79.39 ^{ab}	0.607 ^{bc}	37.814 ^{a}	0.240 ^{bc}	0.163 ^{a}	19.62 ^{b}	11.11 ^{abc}	2.61 ^{cde}	30.73 ^{ab}
	77/0.1 2iP	1.92 ^{cd}	0.48 ^{d}	16.85 ^{c}	0.862 ^{a}	0.000 ^{b}	0.189 ^{c}	0.025 ^{b}	17.49 ^{b}	2.50 ^{c}	8.25 ^{ab}	19.99 ^{bc}
	77/0.1 BAP	1.61 ^{cd}	1.58 ^{abc}	57.27 ^{abc}	0.876 ^{a}	0.000 ^{b}	0.152 ^{c}	0.019 ^{b}	16.58 ^{b}	7.66 ^{abc}	3.60 ^{cde}	24.25 ^{bc}
	77/1	4.83 ^{a}	2.37 ^{a}	78.68 ^{ab}	0.474 ^{c}	37.059 ^{a}	0.356 ^{ab}	0.231 ^{a}	14.97 ^{b}	14.85 ^{a}	0.96 ^{de}	29.82 ^{ab}
	77/1 2iP	2.64 ^{bc}	1.97 ^{a}	83.79 ^{a}	0.865 ^{a}	0.000 ^{b}	0.405 ^{a}	0.054 ^{b}	33.07 ^{a}	7.02 ^{abc}	9.16 ^{a}	40.09 ^{a}
	77/1 BAP	1.57 ^{cd}	0.69 ^{bcd}	31.78 ^{abc}	0.885 ^{a}	0.000 ^{b}	0.135 ^{c}	0.015 ^{b}	13.50 ^{b}	0.85 ^{c}	5.74 ^{abc}	14.35 ^{c}
	Control 2iP	2.12 ^{bcd}	0.43 ^{d}	11.04 ^{c}	0.804 ^{a}	0.591 ^{b}	0.186 ^{c}	0.049 ^{b}	15.49 ^{b}	3.57 ^{bc}	4.92 ^{bcd}	19.06 ^{bc}
	Control BAP	1.60 ^{cd}	1.31 ^{abcd}	60.39 ^{abc}	0.881 ^{a}	0.000 ^{b}	0.147 ^{c}	0.018 ^{b}	15.54 ^{b}	0.07 ^{c}	7.92 ^{ab}	15.61 ^{bc}
Villafranca	52/0.1	6.93 ^{a}	5.57 ^{a}	100.00 ^{a}	0.832 ^{ab}	100.000 ^{c}	0.147 ^{cd}	0.025 ^{b}	4.77 ^{d}	11.76 ^{abc}	0.00 ^{f}	16.53 ^{defg}
	52/0.1 2iP	1.78 ^{ef}	0.32 ^{fg}	44.35 ^{abcd}	0.878 ^{ab}	1.952 ^{c}	0.095 ^{d}	0.013 ^{b}	15.33 ^{cd}	6.85 ^{abc}	3.85 ^{bcdef}	22.18 ^{cdefg}
	52/0.1 BAP	1.47 ^{ef}	0.71 ^{efg}	44.66 ^{abcd}	0.894 ^{ab}	0.000 ^{c}	0.097 ^{d}	0.011 ^{b}	14.32 ^{cd}	0.00 ^{c}	7.13 ^{abcde}	14.32 ^{fg}
	52/1	4.44 ^{bc}	3.23 ^{bc}	95.85 ^{ab}	0.864 ^{ab}	96.985 ^{c}	0.136 ^{d}	0.018 ^{b}	5.00 ^{d}	9.22 ^{abc}	0.00 ^{f}	14.23 ^{fg}
	52/1 2iP	1.94 ^{def}	0.92 ^{defg}	44.35 ^{abcd}	0.868 ^{ab}	0.000 ^{c}	0.121 ^{d}	0.016 ^{b}	26.27 ^{abcd}	12.22 ^{abc}	5.42 ^{abcdef}	38.48 ^{abcde}
	52/1 BAP	1.45 ^{ef}	0.85 ^{efg}	46.34 ^{abcd}	0.880 ^{ab}	0.000 ^{c}	0.092 ^{d}	0.011 ^{b}	18.68 ^{bcd}	0.00 ^{c}	8.35 ^{abc}	18.68 ^{defg}
	77/0.1	4.94 ^{bc}	2.36 ^{bcde}	93.30 ^{ab}	0.737 ^{ab}	79.734 ^{c}	0.072 ^{d}	0.062 ^{b}	5.51 ^{d}	10.40 ^{abc}	0.00 ^{f}	15.92 ^{fg}
	77/0.1 2iP	2.33 ^{def}	0.32 ^{fg}	23.03 ^{bcd}	0.896 ^{ab}	0.000 ^{c}	0.170 ^{cd}	0.017 ^{b}	19.76 ^{bcd}	0.00 ^{c}	10.66 ^{a}	19.76 ^{defg}
	77/0.1 BAP	1.63 ^{ef}	1.71 ^{cdef}	62.04 ^{abcd}	0.882 ^{ab}	0.000 ^{c}	0.109 ^{d}	0.013 ^{b}	10.50 ^{cd}	12.60 ^{abc}	0.00 ^{f}	23.10 ^{bcdefg}
	77/1	5.32 ^{b}	4.22 ^{ab}	96.98 ^{ab}	0.662 ^{bc}	93.301 ^{c}	0.146 ^{cd}	0.052 ^{b}	5.96 ^{d}	10.23 ^{abc}	0.00 ^{f}	16.19 ^{efg}
	77/1 2iP	3.30 ^{cde}	2.68 ^{bcd}	96.51 ^{ab}	0.878 ^{ab}	0.000 ^{c}	0.458 ^{ab}	0.059 ^{b}	37.43 ^{ab}	6.41 ^{abc}	10.83 ^{a}	43.84 ^{ab}
	77/1 BAP	1.79 ^{ef}	1.00 ^{defg}	35.73 ^{bcd}	0.896 ^{ab}	0.000 ^{c}	0.137 ^{d}	0.015 ^{b}	11.78 ^{cd}	0.47 ^{c}	5.97 ^{abcde}	12.25 ^{g}
	Control 2iP	1.64 ^{ef}	0.00 ^{g}	0.00 ^{d}	0.953 ^{a}	0.000 ^{c}	0.103 ^{d}	0.005 ^{b}	15.14 ^{cd}	1.41 ^{c}	6.62 ^{abcde}	16.55 ^{defg}
	Control BAP	1.65 ^{ef}	1.13 ^{defg}	53.35 ^{abcd}	0.882 ^{ab}	0.000 ^{c}	0.093 ^{d}	0.011 ^{b}	16.81 ^{bcd}	0.00 ^{c}	9.60 ^{ab}	16.81 ^{defg}
L-12	52/0.1	2.04 ^{def}	0.08 ^{g}	3.02 ^{cd}	0.494 ^{cd}	0.000 ^{a}	0.211 ^{cd}	0.116 ^{b}	18.65 ^{bcd}	20.09 ^{a}	1.18 ^{ef}	38.75 ^{abcd}
	52/0.1 2iP	1.59 ^{ef}	1.00 ^{defg}	36.17 ^{bcd}	0.822 ^{ab}	0.000 ^{c}	0.169 ^{cd}	0.032 ^{b}	22.92 ^{abcd}	4.11 ^{abc}	5.00 ^{abcdef}	27.03 ^{bcdefg}
	52/0.1 BAP	1.23 ^{f}	0.19 ^{fg}	5.28 ^{cd}	0.871 ^{ab}	0.000 ^{c}	0.153 ^{cd}	0.020 ^{b}	17.15 ^{bcd}	2.73 ^{bc}	5.34 ^{abcdef}	19.88 ^{defg}
	52/1	1.99 ^{def}	0.56 ^{fg}	53.29 ^{abcd}	0.422 ^{d}	0.000 ^{a}	0.251 ^{cd}	0.127 ^{b}	23.25 ^{abcd}	19.35 ^{ab}	6.26 ^{abcde}	42.60 ^{abc}
	52/1 2iP	1.37 ^{f}	0.36 ^{fg}	35.28 ^{bcd}	0.843 ^{ab}	0.000 ^{c}	0.207 ^{cd}	0.031 ^{b}	15.47 ^{cd}	3.32 ^{abc}	3.37 ^{cdef}	18.79 ^{defg}
	52/1 BAP	1.28 ^{f}	0.24 ^{fg}	5.12 ^{cd}	0.879 ^{ab}	0.000 ^{c}	0.115 ^{d}	0.014 ^{b}	15.26 ^{cd}	2.62 ^{bc}	8.28 ^{abc}	17.88 ^{defg}
	77/0.1	3.67 ^{bcd}	0.87 ^{efg}	50.00 ^{abcd}	0.412 ^{d}	0.000 ^{b}	0.491 ^{ab}	0.314 ^{a}	40.78 ^{a}	12.16 ^{abc}	6.52 ^{abcde}	52.94 ^{a}
	77/0.1 2iP	1.51 ^{ef}	0.64 ^{fg}	11.46 ^{cd}	0.829 ^{ab}	0.000 ^{c}	0.208 ^{cd}	0.034 ^{b}	15.22 ^{cd}	4.99 ^{abc}	5.84 ^{abcdef}	20.22 ^{defg}
	77/0.1 BAP	1.58 ^{ef}	1.38 ^{defg}	50.00 ^{abcd}	0.867 ^{ab}	0.000 ^{c}	0.216 ^{cd}	0.028 ^{b}	25.70 ^{abcd}	0.27 ^{c}	9.01 ^{abc}	25.97 ^{bcdefg}
	77/1	4.33 ^{bc}	0.92 ^{defg}	50.00 ^{abcd}	0.286 ^{d}	0.000 ^{ab}	0.566 ^{a}	0.409 ^{a}	23.97 ^{abcd}	19.47 ^{ab}	1.91 ^{def}	43.44 ^{abc}
	77/1 2iP	1.97 ^{def}	1.33 ^{defg}	64.27 ^{abc}	0.851 ^{ab}	0.000 ^{c}	0.351 ^{bc}	0.050 ^{b}	28.71 ^{abc}	7.62 ^{abc}	7.49 ^{abcd}	36.33 ^{abcdef}
	77/1 BAP	1.25 ^{f}	0.28 ^{fg}	26.10 ^{bcd}	0.869 ^{ab}	0.000 ^{c}	0.130 ^{d}	0.017 ^{b}	16.07 ^{bcd}	1.43 ^{c}	5.41 ^{abcdef}	17.50 ^{defg}
	Control 2iP	2.42 ^{def}	0.72 ^{efg}	26.61 ^{bcd}	0.715 ^{ab}	1.508 ^{c}	0.236 ^{cd}	0.076 ^{b}	15.70 ^{cd}	4.86 ^{abc}	3.90 ^{bcdef}	20.57 ^{defg}
	Control BAP	1.52 ^{ef}	1.60 ^{cdef}	70.50 ^{abc}	0.880 ^{ab}	0.000 ^{c}	0.229 ^{cd}	0.027 ^{b}	13.63 ^{cd}	0.17 ^{c}	5.40 ^{abcdef}	13.80 ^{g}

1) Labels of the characteristics: SH - Height of the dominant shoot [cm], MR - Multiplication rate, MP - Multiplication percentage [%], MC - Moisture content [g H_2O g^{-1} DW], RP – Rooting percentage [%], SFW - shoot fresh weight [g $plant^{-1}$], SDW - shoot dry weight [g $plant^{-1}$], Chl a - Chl a content [mg g^{-1} FW], Chl b - Chl b content [mg g^{-1} FW], Car - Carotenoid content [mg g^{-1} FW], Chl a+b - Chl a+b content [mg g^{-1} FW].

2) Same letters between two treatments of the same effect denote differences that are not significant according to Duncan's test for α=0.05.

[3] Labels for formulations: The first number represents the CKX inhibitor, second its medium concentration in μM, and the third label represents presence of BAP or 2iP in medium concentration of 1 μM. Control media has no CKX inhibitor.
