# Supplementary material for: CKX Inhibitors: From Shoot Tip Micropropagation to Rooted Plantlet
Source: Plants (Basel). 2026 Oct 25;15(21):3293. doi: 10.3390/plants15213293 (PMC13765872; doi:10.3390/plants15213293)
Supplement: Supplementary file 1 [file plants-15-03293-s001.zip › Table S2 Results of Duncan’s test for phenolic compound content and antioxidant capacity.pdf]

Table S2. Results of Duncan's test for phenolic compound content and antioxidant capacity.

Clone	Formulation [3]	TPC [1]		TFC		DPPH		ABTS		FRAP	
Villafranca		1.265	b [2]	0.278	b	15.335	a	6.792	b	2.946	b
L-12		1.604	a	0.361	a	13.873	b	7.057	a	4.904	a
	52/0.1	1.870	b	0.224	d	15.223	c	7.156	b	5.511	a
	52/0.1 2iP	1.426	cd	0.357	bc	3.785	d	8.043	a	4.404	bc
	52/0.1 BAP	1.350	cd	0.357	bc	2.754	d	7.981	a	4.559	b
	52/1	1.423	cd	0.270	cd	20.537	ab	6.391	cde	3.264	def
	52/1 2iP	1.192	def	0.343	bc	19.592	ab	6.051	f	2.508	f
	52/1 BAP	1.307	de	0.332	bc	20.363	ab	6.401	cd	2.911	ef
	77/0.1	1.304	de	0.274	cd	19.331	ab	6.153	def	2.852	ef
	77/0.1 2iP	1.143	def	0.472	a	3.142	d	8.016	a	4.371	bc
	77/0.1 BAP	0.872	f	0.392	ab	2.758	d	7.900	a	4.019	bcd
	77/1	1.461	cd	0.280	cd	20.752	ab	6.553	c	3.506	de
	77/1 2iP	1.682	bc	0.333	bc	20.128	ab	6.233	def	3.168	def
	77/1 BAP	2.534	a	0.276	cd	19.939	ab	7.100	b	5.896	a
	Control 2iP	0.989	ef	0.279	cd	18.333	b	6.078	ef	2.932	ef
	Control BAP	1.219	de	0.300	bcd	21.355	a	6.256	cdef	3.617	cde
Villafranca	52/0.1	1.547	defg	0.259	ef	21.801	ab	6.453	ef	4.426	cd
	52/0.1 2iP	0.848	ijk	0.215	f	3.153	e	7.953	ab	2.650	fghi
	52/0.1 BAP	0.703	jk	0.185	f	2.674	e	7.894	ab	2.298	hi
	52/1	1.316	efghi	0.271	ef	21.452	abc	6.357	ef	2.818	efghi
	52/1 2iP	1.096	fghij	0.310	def	18.241	bc	5.888	gh	2.125	i
	52/1 BAP	1.577	cdef	0.242	ef	19.493	abc	6.420	ef	2.610	fghi
	77/0.1	0.916	ijk	0.279	def	18.486	bc	5.813	h	2.184	i
	77/0.1 2iP	1.054	fghijk	0.448	abc	3.216	e	8.106	a	2.925	efghi
	77/0.1 BAP	0.552	k	0.249	ef	2.648	e	7.837	ab	2.124	i
	77/1	1.700	cde	0.278	def	21.248	abc	6.681	de	3.205	defghi
	77/1 2iP	1.678	cde	0.368	cde	19.348	bc	6.060	fgh	2.572	fghi
	77/1 BAP	2.415	ab	0.251	ef	20.261	abc	7.122	c	4.789	bc
	Control 2iP	0.974	ijk	0.290	def	18.999	bc	6.121	fgh	2.463	ghi
	Control BAP	1.331	efghi	0.252	ef	23.671	a	6.388	ef	4.054	cde
L-12	52/0.1	2.064	bc	0.204	f	11.276	d	7.578	b	6.162	a
	52/0.1 2iP	2.004	bcd	0.499	abc	4.417	e	8.134	a	6.158	a
	52/0.1 BAP	1.996	bcd	0.529	ab	2.835	e	8.069	a	6.821	a
	52/1	1.531	defgh	0.270	ef	19.622	abc	6.424	ef	3.709	cdefg
	52/1 2iP	1.336	efghi	0.392	bcde	21.619	abc	6.295	efg	3.084	efghi
	52/1 BAP	1.036	ghijk	0.421	abcd	21.233	abc	6.383	ef	3.211	defghi
	77/0.1	1.691	cde	0.269	ef	20.176	abc	6.493	ef	3.519	cdefgh
	77/0.1 2iP	1.231	efghi	0.495	abc	3.068	e	7.925	ab	5.818	ab
	77/0.1 BAP	1.191	efghij	0.536	a	2.868	e	7.963	ab	5.914	ab
	77/1	1.223	efghij	0.282	def	20.256	abc	6.426	ef	3.807	cdef
	77/1 2iP	1.689	cde	0.281	def	21.298	abc	6.493	ef	4.061	cde
	77/1 BAP	2.653	a	0.301	def	19.617	abc	7.078	cd	7.003	a
	Control 2iP	1.013	hijk	0.262	ef	17.334	c	6.013	fgh	3.636	cdefg
	Control BAP	1.051	fghijk	0.371	cde	17.880	bc	6.058	fgh	2.960	efghi

[1] Labels of characteristics: TPC – total content of phenolics [mg GAE g^{-1} FW], TFC – total content of flavonoids [mg QAE g^{-1} FW], DPPH – DPPH radical scavenging capacity [mg TEAC g^{-1} FW], ABTS – ABTS

radical scavenging capacity [mg TEAC g^{-1} FW], FRAP – ferric-reducing antioxidant power [mg TEAC g^{-1} FW]

2) Same letters between two treatments of the same effect denote differences that are not significant according to Duncan's test for α=0.05

3) Labels for formulations: The first number represents the CKX inhibitor, second its medium concentration in μM, and the third label represents presence of BAP or 2iP in medium concentration of 1 μM. Control media has no CKX inhibitor.
