# Supplementary material for: CKX Inhibitors: From Shoot Tip Micropropagation to Rooted Plantlet
Source: Plants (Basel). 2026 Oct 25;15(21):3293. doi: 10.3390/plants15213293 (PMC13765872; doi:10.3390/plants15213293)
Supplement: Supplementary file 1 [file plants-15-03293-s001.zip › Table S3. Results of Duncan's test for number and length of roots .pdf]

Table S3. Results of Duncan's test for number and length of roots

Clone	Formulation [3]	RN [1]		LR [mm]	
Villafranca		2.017	a[2]	2.427	a
L-12		0.216	b	4.333	a
	52/0.1	2.733	a	5.431	ab
	52/1	1.725	b	2.806	bc
	77/0.1	1.333	b	8.333	a
	77/1	1.092	b	4.379	ab
	Control 2iP	0.000	c	0.000	c
	Control BAP	0.000	c	0.000	c
Villafranca	52/0.1	5.250	a	3.861	bc
	52/1	3.139	b	3.056	bc
	77/0.1	1.778	c	4.222	bc
	77/1	1.933	c	3.425	bc
	Control 2iP	0.000	d	0.000	c
	Control BAP	0.000	d	0.000	c
L-12	52/0.1	0.217	d	7.000	b
	52/1	0.311	d	2.556	bc
	77/0.1	0.667	d	14.500	a
	77/1	0.250	d	5.333	bc
	Control 2iP	0.000	d	0.000	c
	Control BAP	0.000	d	0.000	c

[1] Labels of characteristics: RN – number of roots, LR – length of the longest root,

[2] Same letters between two treatments of the same effect denote differences that are not significant according to Duncan's test for α=0.05,

[3] Labels for formulations: The first number represents the CKX inhibitor, second its medium concentration in μM, and the third label represents presence of BAP or 2iP in medium concentration of 1 μM. Control media has no CKX inhibitor.
